# Neuroimaging evidence of acupuncture in cognitive impairment following ischemic stroke: a systematic review

**DOI:** 10.3389/fnins.2025.1629305

**Published:** 2026-01-12

**Authors:** Chenyang Qin, Bo Li, Bifang Zhuo, Xinming Yang, Ying Cui, Zhihong Meng

**Affiliations:** 1 First Teaching Hospital of Tianjin University of Traditional Chinese Medicine, Tianjin, China; 2 National Clinical Research Center for Chinese Medicine Acupuncture and Moxibustion, Tianjin, China; 3 Tianjin University of Traditional Chinese Medicine, Tianjin, China

**Keywords:** acupuncture, cognitive impairment, neuroimaging, ischemic stroke, MRI

## Abstract

**Objective:**

This review aimed to summarize neuroimaging evidence on the effects of acupuncture in post-ischemic stroke cognitive impairment (PISCI) and to explore its potential neural mechanisms.

**Methods:**

A systematic search was conducted across multiple databases, including China National Knowledge Infrastructure (CNKI), SinoMed (China Biology Medicine Disc), the Chinese Scientific Journal Database (VIP), Wanfang Data, PubMed, the Cochrane Library, Embase, and Web of Science. Studies were selected according to inclusion and exclusion criteria. Risk of bias was assessed for all eligible studies.

**Results:**

Eight studies met the inclusion criteria. These studies utilized resting-state functional magnetic resonance imaging (rs-fMRI) and magnetic resonance spectroscopy (MRS) to investigate the effects of acupuncture on brain activity and metabolic changes. The neuroimaging findings showed that all studies focused on the sustained effects of acupuncture on brain functional activity.

**Conclusions:**

This review provides preliminary neuroimaging evidence supporting the potential benefits of acupuncture for PISCI. The findings suggest that the possible mechanisms of acupuncture for PISCI involve changes in the activity and enhanced functional connectivity of cognition-related brain regions. Additionally, acupuncture may influence brain networks and regulate neurochemical metabolites within cognition-related regions. However, as this field remains in its early stages, further validation is needed. Future studies should focus on well-designed, multicenter randomized controlled trials (RCTs) with large sample sizes and incorporate multiple neuroimaging techniques to better clarify and verify the neural mechanisms of acupuncture in PISCI.

**Systematic review registration:**

PROSPERO, identifier: CRD420250652194.

## 1 Introduction

Each year, approximately 12.2 million people are newly diagnosed with stroke worldwide. Among adults, stroke is the leading cause of long-term disability and the second leading cause of death globally (Hilkens et al., 2024; Lekoubou et al., 2023). According to the 2021 Global Burden of Disease (GBD) study, stroke represents the most burdensome disease across all regions of East Asia (Ferrari et al., 2024). Ischemic stroke accounts for approximately 87% of all stroke cases (Li Y. et al., 2023) and is primarily caused by arterial occlusion leading to focal cerebral ischemia and hypoxia, which subsequently result in neuronal death and disruption of neural networks (Guo et al., 2021). Despite advances in acute treatments such as thrombolysis and mechanical thrombectomy, a significant proportion of stroke survivors experience varying degrees of cognitive impairment during recovery (Huang et al., 2022; Kalaria et al., 2016; Li et al., 2022). Post-stroke cognitive impairment (PSCI) is a common complication characterized by memory decline, reduced attention, and executive dysfunction, which significantly affect patients' quality of life and life expectancy (Stinear et al., 2020). The prevalence and incidence of PSCI vary depending on the outcome definition and the timing of assessment (Pendlebury and Rothwell, 2009). A large review of almost 300,000 individuals in 12 countries indicated that the prevalence of PSCI ranges from 20% to 80% (Sun et al., 2014). Among its subtypes, post-ischemic stroke cognitive impairment (PISCI) is a major form of PSCI and has been associated with adverse outcomes, including severe disability, depression, increased mortality, and recurrent strokes (Korostynski et al., 2021; Kwon et al., 2020; Mijajlović et al., 2017; Swartz et al., 2016). However, effective treatment options for PISCI remain limited.

Current therapeutic strategies for PISCI primarily include cholinesterase inhibitors, cognitive training, and non-invasive brain stimulation techniques (Cicerone et al., 2011; Wang et al., 2022c; Whyte et al., 2008; Winstein et al., 2016). However, pharmacological treatments often provide only temporary symptomatic relief, do not substantially delay disease progression, and are frequently associated with gastrointestinal side effects (Beristain and Golombievski, 2015; Eshaghi Ghalibaf et al., 2023; Malik et al., 2022). The long term effectiveness of cognitive training is constrained by patient adherence and the need for individualized interventions (Beristain and Golombievski, 2015; Irazoki et al., 2020). Additionally, the clinical efficacy of non-invasive brain stimulation remains inconclusive (Snowball et al., 2013; Yang et al., 2024). Therefore, there is an urgent need to explore safe and effective alternative or adjunctive therapies. Acupuncture, a core treatment modality in Traditional Chinese Medicine (TCM), is widely applied in stroke rehabilitation (Liu et al., 2021; Qiu et al., 2021; Wang et al., 2022a; Yang et al., 2022) and has shown promising effects in improving post-stroke cognitive function (Chavez et al., 2017). Several meta-analyses suggest potential benefits of acupuncture for PISCI (Han et al., 2024; Kuang et al., 2021; Liu et al., 2023; Wang et al., 2022d; Wu et al., 2024). For example, Su et al. (2024) reported that acupuncture combined with repetitive transcranial magnetic stimulation (rTMS) was more effective than rTMS alone, while Liu et al. (2023) found that acupuncture was more effective than conventional rehabilitation training in improving cognitive function. In addition, a multicenter RCT (Zhang et al., 2022) indicated that acupuncture significantly improved post-stroke cognitive function.

Nevertheless, the mechanisms by which acupuncture promotes cognitive recovery remain unclear. Several studies have provided insights into the potential mechanisms by which acupuncture ameliorates PISCI. A review suggested that acupuncture may enhance synaptic plasticity by regulating long-term potentiation (LTP) and long-term depression (LTD), thereby facilitating cognitive recovery (Qin et al., 2022). Other studies have shown that acupuncture can inhibit neuronal apoptosis (Li N. et al., 2023), a pathological process considered to be a key contributor to cognitive decline after stroke (Mattson, 2000). In addition, electro acupuncture stimulation has been reported to suppress the expression of inflammatory cytokines in the hippocampus and plasma, leading to improved cognitive function in rats (Wang et al., 2020). Studies have also demonstrated that acupuncture may alleviate cognitive impairment by enhancing cerebral blood flow (Ma et al., 2020) and reducing oxidative stress (Du et al., 2018). Collectively, these findings suggest that acupuncture exerts regulatory effects through multiple neurobiological mechanisms, although the specific processes remain to be fully elucidated.

PISCI has been linked to both functional and structural alterations in multiple brain regions, including disrupted functional connectivity (Bournonville et al., 2018), structural changes (Benedict et al., 2020), abnormal neural network activity (Wahl, 2018), and neuro inflammatory responses (Shishkina et al., 2021), all of which contribute to cognitive decline. Neuroimaging techniques, such as functional magnetic resonance imaging (fMRI), diffusion tensor imaging (DTI), functional near-infrared spectroscopy (fNIRS), and magnetic resonance spectroscopy (MRS), provide crucial tools for investigating the neural mechanisms underlying PISCI (Yu et al., 2024) and offer novel perspectives for exploring the effects of acupuncture. To date, however, no systematic review has comprehensively synthesized neuroimaging evidence on the effects of acupuncture in PISCI. Accordingly, the present review summarizes current neuroimaging findings on acupuncture in PISCI and explores potential neural mechanisms. Through this synthesis, the review seeks to deepen understanding of how acupuncture may improve cognitive outcomes in PISCI and to provide a theoretical foundation for its clinical application.

## 2 Methods

### 2.1 Protocol and registration

The protocol for this systematic review (SR) was registered in PROSPERO (registration number CRD420250652194). The review was conducted in accordance with the PRISMA statement (Moher et al., 2009).

### 2.2 Eligibility criteria

#### 2.2.1 Inclusion criteria

The inclusion criteria are shown in Table 1.

**Table 1:** Inclusion criteria of this study.

Study type	Published randomized and non-randomized controlled studies (RCTs and non-RCTs) in English and Chinese on acupuncture for PISCI.
Patients	Meeting the diagnostic criteria for ischemic stroke, with cognitive impairment confirmed by neuropsychological assessments, and no restrictions on age, gender, race, or region.
Intervention	(1) studies in which acupuncture was the sole intervention in the treatment group, with the control group receiving either a placebo, drug, conventional therapy, or sham acupuncture; or (2) studies where acupuncture was administered alongside other therapies in the treatment group, provided that the control group received those same other therapies without the acupuncture.
Outcomes	At least one of the following neuroimaging tools was used: MRI, DTI, MRS, or fNIRS.

#### 2.2.2 Exclusion criteria

Studies were excluded if they met any of the following conditions:

(1) Reviews, comments, animal studies, letters, protocols, case reports, or duplicate studies;
(2) Studies involving non-ischemic stroke populations or without a clear assessment of cognitive impairment;
(3) Studies comparing two different acupuncture methods;
(4) Studies lacking available data on both neuroimaging outcomes and cognitive function outcomes;
(5) Studies not published in Chinese or English, or studies for which the full text was unavailable.

### 2.3 Information sources and search strategy

A systematic search was conducted across multiple databases from their inception to February 28, 2025. The databases included China National Knowledge Infrastructure (CNKI), SinoMed (China Biology Medicine Disc), the Chinese Scientific Journal Database (VIP), Wanfang Data, PubMed, the Cochrane Library, Embase, and Web of Science. The main search terms are listed in Table 2.

**Table 2:** The main terms of this study.

**Number**	**Terms**
1	Cognitive dysfunction
2	Cognitive impairment
3	Cognitive decline
4	Acupuncture
5	Acupoint
6	Needle
7	Warm needling
8	Warm acupuncture
9	Electronic acupuncture
10	Electro acupuncture
11	Fire acupuncture
12	Auricular needle
13	Scalp needle
14	Abdominal needle
15	Wrist ankle needle
16	Body acupuncture
17	Ischemic stroke
18	Apoplexy
19	Brain infarction
20	Cerebral infarction
21	Cerebrovascular accident
22	Cerebrovascular apoplexy
23	Neuroimaging
24	MRI
25	Magnetic Resonance Imaging
26	PET
27	Positron-Emission Tomography
28	fMRI
29	Functional magnetic resonance imaging
30	DTI
31	Diffusion Tensor Imaging
32	sMRI
33	Structural magnetic resonance imaging
34	fNIRS
35	Functional near-infrared spectroscopy
36	MRS
37	Magnetic resonance spectroscopy
38	Cognitive impairment-related terms: 1 OR 2 OR 3
39	Acupuncture-related terms: 4 OR 5 OR 6 OR 7 OR 8 OR 9 OR 10 OR 11 OR 12 OR 13 OR 14 OR 15 OR 16
40	Stroke-related terms: 17 OR 18 OR 19 OR 20 OR 21 OR 22
41	Neuroimaging-related terms: 23 OR 24 OR 25 OR 26 OR 27 OR 28 OR 29 30 OR 31 OR 32 OR 33 OR 34 OR 35 OR 36 OR 37
42	Final searching terms: 38 AND 39 AND 40 AND 41

### 2.4 Selection process

The retrieved articles were first imported into NoteExpress software (version 4.1.0) to remove duplicates. Two independent researchers (Qin and Zhuo) then screened titles and abstracts based on the eligibility criteria. Full texts of potentially eligible studies were subsequently reviewed to determine final inclusion. Any discrepancies were resolved through consensus.

### 2.5 Data collection process

Data extraction was carried out by Yang using a standardized template in Microsoft Excel 2019 to record the following information: general study characteristics (first author, year of publication, study design), participant details (sample size, sex, age, time since stroke, treatment duration), intervention information, control group details, and outcome measures. The Standards for Reporting Interventions in Clinical Trials of Acupuncture (STRICTA) were applied to ensure comprehensive reporting of acupuncture details (e.g., needle depth, retention time). Discrepancies were resolved through discussion among all researchers.

### 2.6 Quality assessment

Two researchers (Qin and Li) assessed the methodological quality of RCTs using the Cochrane Risk of Bias 2.0 tool (RoB 2). The RoB results were classified as high risk, low risk, or some concerns. Disagreements were resolved through consensus.

### 2.7 Synthesis methods

Due to methodological heterogeneity among the included studies, a descriptive analysis was conducted. The synthesis aimed to provide neuroimaging evidence supporting the clinical efficacy of acupuncture for PISCI.

## 3 Results

### 3.1 Study selection

**Figure 1:**
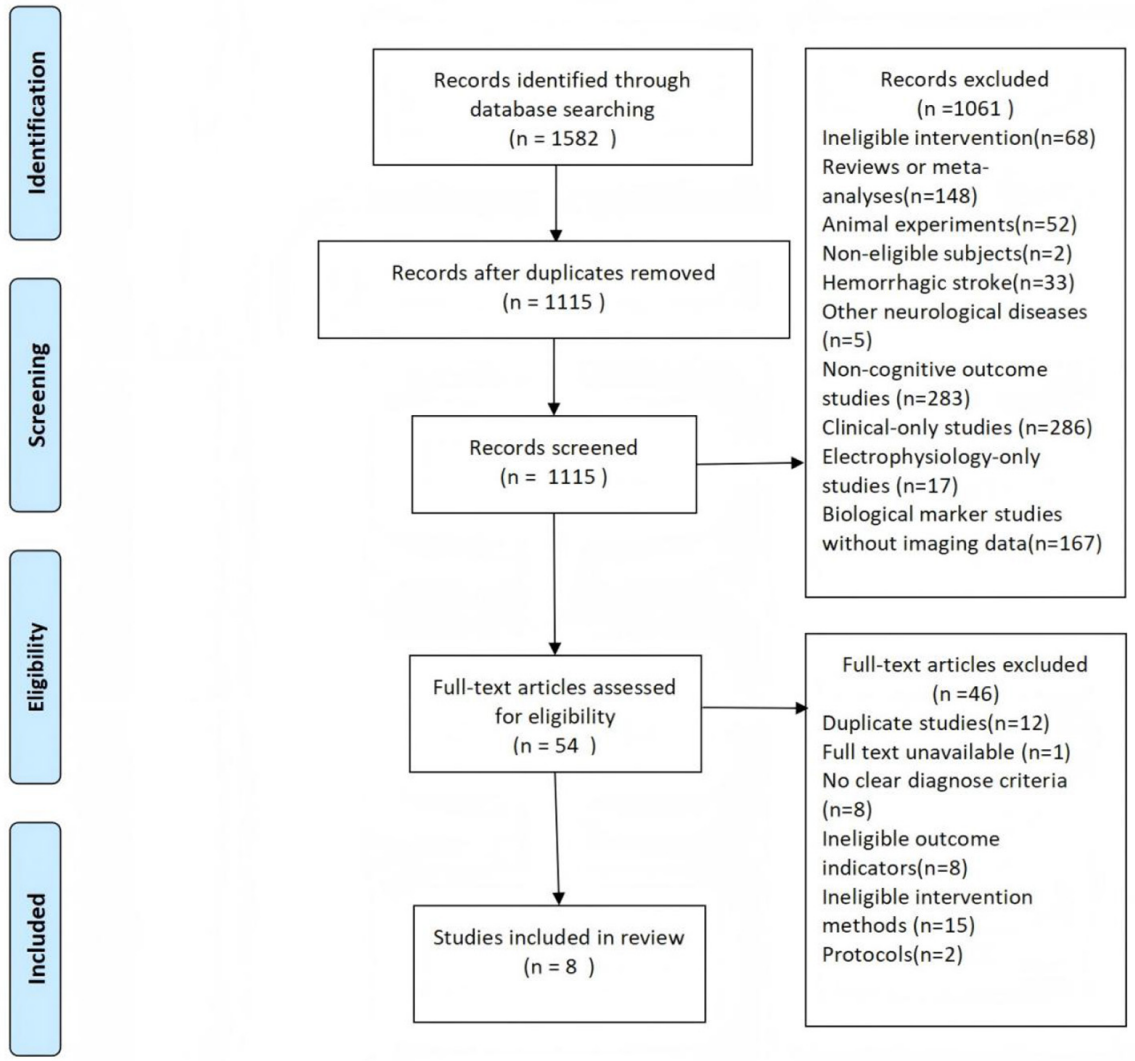
Flow chart of selection process.

A comprehensive search across multiple databases initially retrieved 1,582 records. After removing duplicates using reference management software, 1,115 records remained. Titles, abstracts, and keywords were then screened against the eligibility criteria, yielding 54 potentially relevant studies. Following full-text assessment, 8 studies (Li, 2023; Su, 2016; Wang et al., 2014, 2021; Wang, 2021; Xiao and Yu, 2024; Yu et al., 2021; Zhang et al., 2020) were included for analysis. The PRISMA flowchart of the search and selection process is shown in Figure 1.

### 3.2 Study characteristics

Table 3 summarizes characteristics of the 8 included studies, published between 2014 and 2024. These studies comprised 5 RCTs and 3 non-RCTs, all conducted in China.

**Table 3:** Characteristics of studies included in the systematic review.

**Study**	**Study type**	**Year**	**Sample Size (T/C)**	**Gender (%males)**	**Age (years)**	**Time since stroke**	**Treatment courses**	**Type of T patient**	**Type of C patient**	**Intervention (T)**	**Intervention (C)**	**Imaging modality**	**Clinical outcomes**	**Analytical approaches**	**Scan T**
Wei Xiao	RCT	2024	25(17/8)	52%	T: 63.00 ± 6.34 C: 58.25 ± 6.60	Within 3 months	2 weeks	PISCI	PISCI	Acupuncture	Sham acupuncture	rs-fMRI	MMSE, MoCA	DC	/
Xiayu Li	Non-RCT	2023	32(16/16)	75%	T: 60.5 ± 1.2 C: 61.5 ± 1.2	Within 6 months	12 weeks	PISCI	HC	“Regulating Mind and Enlightening Wisdom” acupuncture	/	rs-fMRI	MMSE, MoCA, WMS-RC	FALFF, ReHo, DC	3.0 T
Fei Wang	RCT	2021	80(40/40)	63%	T: 66 ± 8 C: 67 ± 9	Within 30 days	8 weeks	PISCI	PISCI	Acupuncture and Cognitive Training	Cognitive Training	rs-fMRI	MMSE, MoCA, NIHSS, FMA, MBI	FC (ROI)	3.0 T
Ran Wang	Non-RCT	2021	36(22/14)	42%	T: 60.14 ± 8.46 C: 56.71 ± 4.68	/	2 weeks	PISCI	HC	Acupuncture	/	rs-fMRI	MMSE, MoCA	FC (DC, local efficiency)	3.0T
Yanli Yu	RCT	2021	60(30/30)	58%	T: 59 ± 3 C: 59 ± 3	More than 3 weeks	4 weeks	PISCI	PISCI	Bo's Abdominal Acupuncture and Cognitive Training	Cognitive Training	rs-fMRI	MoCA, TMT, AVLT, DS	ALFF	/
Jianbo Zhang	RCT	2020	60(30/30)	63%	T: 70.10 ± 4.51 C: 69.03 ± 4.70	More than 2 weeks	6 weeks	PISCI	PISCI	Scalp electro acupuncture and Computer-Assisted Training	Computer-Assisted Training	MRS	MoCA	Metabolic Ration	3.0T
Mengrun Sun	Non-RCT	2016	60(30/30)	52%	T: 68.23 ± 5.99 C: 70.87 ± 7.32	Within 2 weeks	4 weeks	PISCI	PISCI	Acupuncture and nimodipine	Nimodipine	MRS	MMSE, MoCA	Metabolic Ration	3.0T
Fang Wang	RCT	2014	60(30/30)	52%	45-80	3-6 months	12 weeks	PISCI	PISCI	Acupuncture and nimodipine	Nimodipine	MRS	MoCA	Metabolic Ration	3.0 T

T, treatment group; C, control group; HC, healthy control; MMSE, Mini-Mental State Examination; MoCA, Montreal Cognitive Assessment; WMS-RC, Wechsler Memory Scale-Revised in China; AVLT, auditory verbal learning test; DS, digital span; TMT, Trail Making Test; FC: functional connectivity; DC: degree centrality, FALFF, fractional amplitude of low frequency fluctuation; ReHo, regional homogeneity; ALFF, amplitude of low-frequency fluctuation; ROI: region of interest; rs-fMRI, resting-state functional magnetic resonance imaging; NIHSS, National Institute of Health stroke scale; FMA, Fugl-Meyer assessment; MBI, modified Barthel index.

### 3.3 Quality assessment

**Figure 2:**
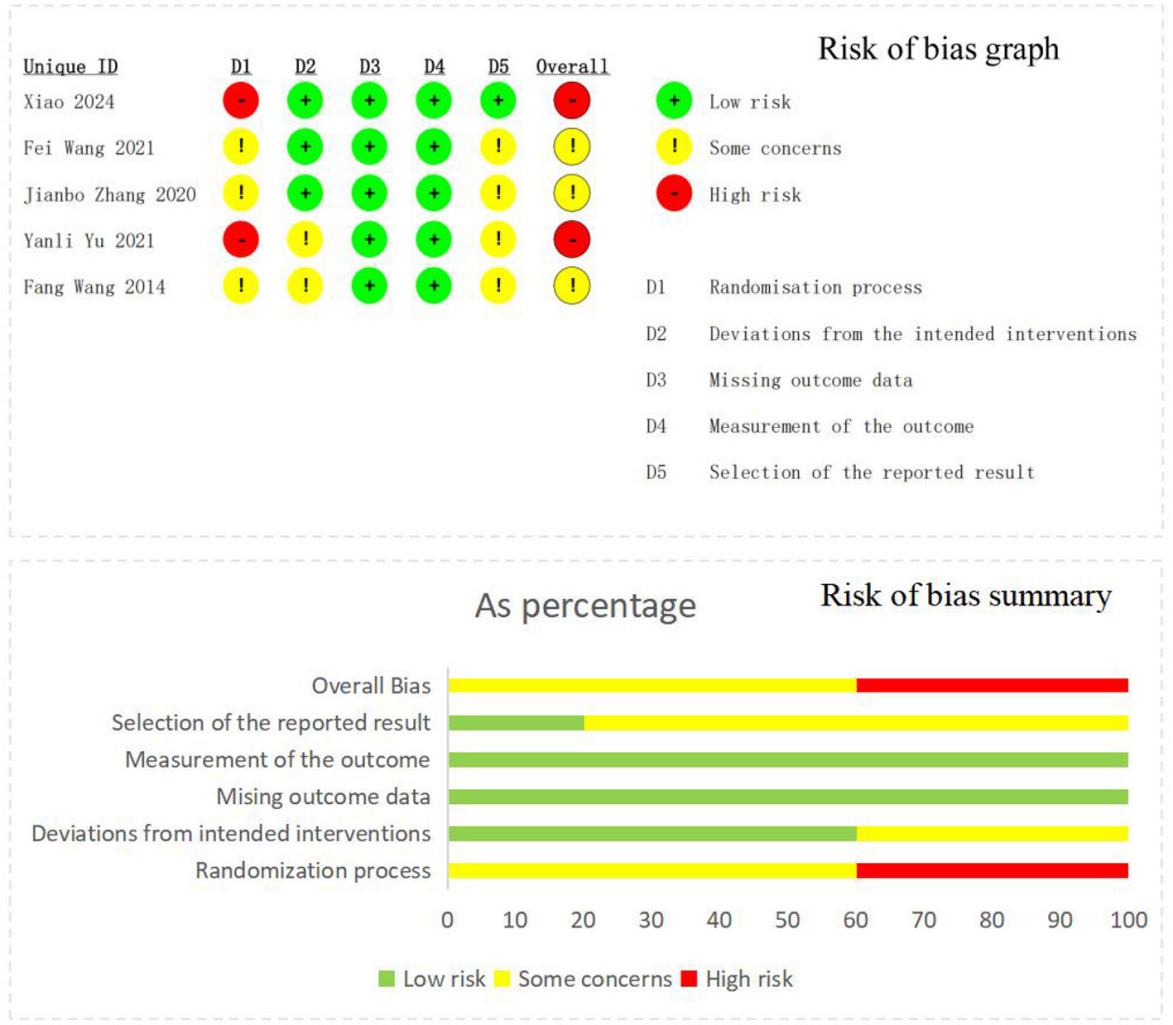
Risk of bias graph and summary.

The risk of bias assessment for the included RCTs is presented in Figure 2. Of the five RCTs, two were judged to have a high risk of bias, and three were rated as having some concerns according to the RoB 2 tool. Regarding the randomization process, all studies presented certain issues due to inadequate reporting of random sequence generation or allocation concealment. With respect to deviations from the intended interventions, none of the studies blinded acupuncturists, which is expected given the nature of the intervention. Three studies reported participant blinding and were rated as low risk; the remaining two raised some concerns due to unclear blinding descriptions. Risk of bias due to missing outcome data was generally low across all studies. Measurement of the outcome was rated as low risk in all studies. In terms of the selection of the reported result, potential bias may exist due to the lack of complete study protocols or trial registration information.

### 3.4 Participants

A total of 397 participants were included across the eight studies, comprising 367 patients with PISCI and 30 healthy controls. Sample sizes ranged from 25 to 80. Two studies compared PISCI patients with healthy controls, while six enrolled only PISCI patients. The age of PISCI patients ranged from 45 to 80 years. All studies reported gender; in total, there were 237 males and 150 females, and only one study had a male proportion below 50%.

### 3.5 Intervention

**Figure 3:**
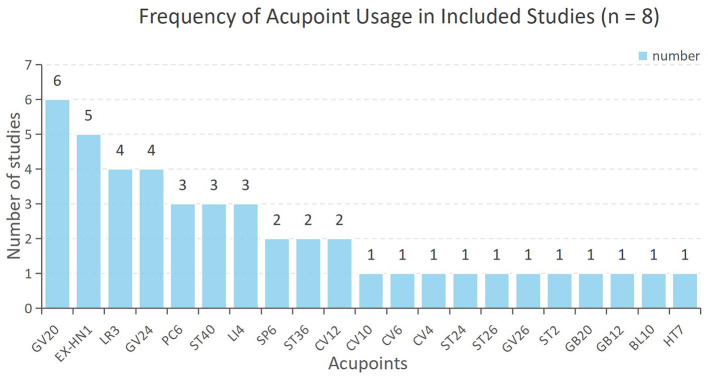
Frequency of acupoint usage in included studies (*n* = 8).

In accordance with STRICTA, acupuncture details are summarized in Table 4. All studies reported the theoretical rationale for acupoint selection. The number of needles used per session ranged from 1 to 22 across studies. Among all studies, GV20 (6/8, 75%) was the most frequently used acupoint, followed by EX-HN1 (5/8, 62.5%), and both GV24 and LR3 (4/8, 50% each) (Figure 3). Acupuncture types included scalp acupuncture (1/8, 12.5%), abdominal acupuncture (1/8, 12.5%), and body acupuncture (6/8, 75%). Manual acupuncture predominated (7/8, 87.5%), while electro acupuncture was used in one study (1/8, 12.5%). Needle insertion depth was reported in six studies (15-50 mm) and a specific needling response (e.g., “Deqi”) was described in five studies (5/8, 62.5%). Except for one study, all reported total treatment sessions, ranging from 10 to 72 over 2 to 12 weeks. Practitioner qualifications were reported in three studies (3/8, 37.5%).

**Table 4:** Details of included studies according to Standards for Reporting Interventions in Clinical Trials of Acupuncture (STRICTA).

**Study**	**1.Acupuncture rational**			**2.Details of needling**							**3.Treatment regimen**		**4.Other components of treatment**		**5.Practitioner background**	**6.Control or comparator interventions**	
	**1a**	**1b**	**1c**	**2a**	**2b**	**2c**	**2d**	**2e**	**2f**	**2g**	**3a**	**3b**	**4a**	**4b**	**5**	**6a**	**6b**
Wei Xiao	TCM	Y	NA	7	PC6, GV20, EX-HN1	20-25mm	Deqi	Manual	30min	Needle brand: Jiangsu Medical Supplies Factory Co., Ltd.	NR	Duration: 2 weeks	NR	Y	Y	NR	Y
Xiayu Li	TCM	Y	NA	22	GV20, EX-HN1, GV26, ST2, GB20, GB12, BL10, PC6, HT7, ST40, SP6, LR3	15mm-30mm	NR	Manual	30min	Diameter and length: 0.25mm × 40mm Needle brand: Hwato	36	Frequency: three times per week Duration: 12 weeks	Conventional therapy	NR	NR	Y	Y
Fei Wang	TCM	Y	NA	1	GV20, GV24	20–30mm	Deqi	Manual	30min	Diameter and length: 0.35 × 25mm Needle brand: Hwato	28	Frequency: every other day Duration: 8 weeks	Cognitive Training	NR	NR	Y	Y
Ran Wang	TCM	Y	NA	7	PC6, GV20, EX-HN1	20–25mm	Deqi	Manual	30min	Diameter and length: 0.35 × 50mm	10	Frequency: five times per week Duration:2 weeks	NR	NR	NR	NA	NA
Jianbo Zhang	TCM	Y	NA	5	MS1, MS6 (bilateral)	15-20mm	Deqi	Electronic	30min	Diameter and length: 0.30 × 25mm& 0.25 × 40mm Needle brand: Dongbang Electroacupuncture apparatus: KWD-808I	30	Frequency: five times per week Duration:6weeks	Computer-Assisted Training	NR	NR	Y	Y
Yanli Yu	TCM	Y	NA	NR	CV 12, CV 10, CV 6, CV 4, bilateral ST24, bilateral ST26, bilateral LI,4, bilateral LR3, shangfengshidian, shangfengshishangdian, shangdengshiwaidian, xiafengshidian, and xiafengshixiadian of affected side	NR	NR	Manual	30min	NR	20	Frequency: five times per week Duration: 4 weeks	Cognitive Training	NR	NR	NR	NR
Mengrun Sun	TCM	Y	NA	NR	GV20, GV24, EX-HN1, LR3, LI4, SP6, ST40, ST36	15mm-50mm	Deqi	Manual	30min	Needle brand: Jiajian	24	Frequency: six times per week Duration:4 weeks	Nimodipine	NR	Y	Y	Y
Fang Wang	TCM	Y	NA	NR	GV20, GV24, EX-HN1, LI4, LR3, SP6, ST40, CV12, ST36	NR	NR	Manual	30min	NR	72	Frequency: six times per week Duration:12 weeks	Nimodipine	NR	Y	Y	Y

1a, Style of acupuncture; 1b, Reasoning for provided treatment, based on historical context, literature sources, or consensus methods, with references where appropriate; 1c, Extent to which treatment varied; 2a, Number of needle insertions per subject per session; 2b, Names; 2c, Depth of insertion, based on a specified unit of measurement, or on a particular tissue level; 2d, Response sought; 2e, Needle stimulation; 2f, Needle retention time; 2g, Needle type; 3a, Number of treatment sessions; 3b, Frequency and duration of treatment sessions; 4a, Details of other interventions administered to the acupuncture group; 4b, Setting and context of treatment, including instructions to practitioners, information and explanations to patients; 5, Description of participating acupuncturists; 6a, The rationale for the control or comparator in the context of the research question, with sources that justify this choice; 6b, A precise description of the control or comparator. If sham acupuncture or any other type of acupuncture-like control was used, provide details as in items 1–3 above; TCM, Traditional Chinese Medicine; Y, yes; NA, not application; NR, not reported. MS1, middle line of forehead; MS2, anterior oblique parietotemporal line.

### 3.6 Comparison

Four types of comparisons were identified across the eight studies: acupuncture vs. sham acupuncture (1/8, 12.5%); acupuncture plus drug vs. drug alone (2/8, 25.0%); acupuncture vs. healthy volunteers (2/8, 25.0%); and acupuncture plus cognitive training vs. cognitive training alone (3/8, 37.5%).

### 3.7 Clinical outcomes

PISCI was assessed across multiple domains. All studies used MoCA to evaluate cognitive function, with most (62.5%) also employing MMSE. Additional cognitive measures and assessments of neurological, motor, and daily living functions were used in individual studies (detailed in Table 3).

### 3.8 Neuroimaging results

#### 3.8.1 MRI imaging analysis methods

Five studies (5/8, 62.5%) used rs-fMRI, employing analytical methods including fALFF/ALFF, ReHo, DC, and functional connectivity (FC). Three studies (3/8, 37.5%) used MRS to evaluate metabolic ratios (NAA/Cr, Cho/Cr, MI/Cr). Scanner specifications varied: although two studies (2/8, 25%) did not report the MRI scanner model, the remaining six used 3.0-T scanners from manufacturers including Siemens and GE. Imaging acquisition parameters showed heterogeneity across studies, with TR (repetition time) ranging from 1,500-3,000 ms in rs-fMRI studies and TE (echo time) ranging from 30-40 ms. Preprocessing steps and analytical software also varied, with studies using different sequences and voxel sizes. All studies focused on sustained post-treatment effects of acupuncture. Complete technical specifications are provided in Table 5. Detailed neuroimaging findings are provided in the Supplementary material.

**Table 5:** Imaging parameters of the included studies.

**Study**	**Scanner**	**Field strength**	**Type**	**TR (ms)**	**TE (ms)**	**Sequence**	**Voxel size**	**Key metric**	**Location**
Wei Xiao	NR	NR	rs-fMRI	NR	NR	NR	NR	DC	Whole brain
Xiayu Li	Siemens Skyra	3.0T	rs-fMRI	2000	30	ep2d-bold-rest	3 × 3 × 3 mm3	fALFF, ReHo, DC	Whole brain
Fei Wang	Siemens Skyra	3.0T	rs-fMRI	3000	40	Single-shot EPI	NR	FC (Hippocampus)	Bilateral Hippocampus
Ran Wang	GE MR750	3.0T	rs-fMRI	2000	30	rs-fMRI	3 × 3 × 3 mm3	Graph Theory	Whole brain
Yanli Yu	NR	NR	rs-fMRI	NR	NR	NR	NR	ALFF	Whole brain
Jianbo Zhang	Siemens Prisma	3.0T	MRS	2000	144	PRESS	1 × 1 × 1 cm3	NAA, Cr, Cho, MI	Lesion area
Mengrun Su	Siemens	3.0T	MRS	1500	30	PRESS	2 × 2 × 2 cm	NAA, Cr, Cho, MI	Bilateral frontal white matter
Fang Wang	GE GEMS	3.0T	MRS	NR	NR	PRESS	NR	NAA, Cr, Cho	Hippocampus

NR, not reported; TR, repetition time; TE, echo time; rs-fMRI, resting-state functional magnetic resonance imaging; MRS, magnetic resonance spectroscopy; fALFF, fractional amplitude of low-frequency fluctuation; ALFF, amplitude of low-frequency fluctuation; ReHo, regional homogeneity; DC, degree centrality; FC, functional connectivity; NAA, N-acetylaspartate; Cr, creatine; Cho, choline; MI, myo-inositol; PRESS, point resolved spectroscopy; EPI, echo-planar imaging. Scanner field strength was 3.0 Tesla (T) for all studies where reported.

#### 3.8.2 Acupuncture-induced neuroimaging changes

To assess the consistency of findings across studies, we conducted a systematic synthesis of neuroimaging outcomes across all included studies (Table 6), explicitly indicating the direction of change (increase or decrease) for each metric. Despite heterogeneity in study design and analysis methods, notable consistency emerged in several key regions. Resting-state fMRI demonstrated consistent patterns in several brain regions: bilateral lingual gyrus, bilateral fusiform gyrus, bilateral para hippocampal gyrus (all showing increased activity), and right angular gyrus (decreased activity). MRS findings showed consistency across all three studies, with NAA/Cr ratios consistently increasing and Cho/Cr and MI/Cr consistently decreasing following acupuncture treatment.

**Table 6:** Direction of neuroimaging changes after acupuncture treatment.

**Brain region**	**Metric**	**Studies**	**Direction**
**A. Resting-state fMRI findings**			
L. Parahippocampal gyrus	DC, Local efficiency	Wei Xiao, Ran Wang	↑↑ (2/2)
R. Parahippocampal gyrus	ALFF	Yanli Yu	↑ (1/1)
Hippocampus-prefrontal cortex	FC	Fei Wang	↑ (1/1)
L. Fusiform gyrus	DC, fALFF, Local efficiency	Wei Xiao, Xiayu Li, Ran Wang	↑*↑↑* (3/3)
L. Lingual gyrus	DC	Wei Xiao 2024, Ran Wang	↑↑ (2/2)
R. Lingual gyrus	DC	Wei Xiao, Ran Wang	↑↑ (2/2)
R. Inferior occipital gyrus	DC, Local efficiency	Wei Xiao, Ran Wang	↑↑ (2/2)
L. Posterior cingulate gyrus	DC	Wei Xiao	↑ (1/1)
L. Anterior cingulate cortex	ALFF	Yanli Yu	↑ (1/1)
Transverse temporal gyrus	DC, Local efficiency	Wei Xiao, Ran Wang	↑↑ (2/2)
L. Thalamus	ALFF	Yanli Yu	↑ (1/1)
R. Insula	ALFF	Yanli Yu	↑ (1/1)
R. Superior occipital gyrus	fALFF	Xiayu Li	↓ (1/1)
R. Angular gyrus	DC, Local efficiency	Wei Xiao, Ran Wang	↓↓ (2/2)
L. Inferior frontal gyrus	ALFF	Yanli Yu	↓ (1/1)
L. Inferior temporal gyrus	ALFF	Yanli Yu	↓ (1/1)
R. Inferior temporal gyrus	ALFF	Yanli Yu	↓ (1/1)
L. Middle temporal gyrus	ALFF	Yanli Yu	↓ (1/1)
L. Superior parietal lobule	ALFF	Yanli Yu	↓ (1/1)
L. Posterior cerebellum	ALFF	Yanli Yu	↓ (1/1)
L. Inferior occipital gyrus	DC vs. ALFF	↑: Wei Xiao, Ran Wang; ↓: Yanli Yu	Divergent: ↑↑ (2/3) vs. ↓ (1/3)^*^
**B. Magnetic resonance spectroscopy findings**			
**Metabolite ratio**	**ROI locations**	**Studies**	**Direction**
NAA/Cr	Infarct area, frontal WM, hippocampus	Jianbo Zhang, Mengrun Su, Fang Wang	↑*↑↑* (3/3)
Cho/Cr	Infarct area, hippocampus	Jianbo Zhang, Fang Wang	↓↓ (2/2)
MI/Cr	Infarct area, frontal WM	Jianbo Zhang, Mengrun Su	↓↓ (2/2)

L, left; R, right. ↑ indicates increased activity; ↓ indicates decreased activity. Numbers in parentheses indicate the number of studies showing the finding (e.g., ↑*↑↑* (3/3) means 3 out of 3 studies showed an increase). ^*^Indicates divergent findings: DC showed increased activity in 2 studies, while ALFF showed decreased activity in 1 study. ALFF, amplitude of low-frequency fluctuation; fALFF, fractional ALFF; DC, degree centrality; FC, functional connectivity. WM, white matter; NAA/Cho/MI, N-acetylaspartate/choline/myo-inositol; Cr, creatine.

##### 3.8.2.1 Acupuncture-induced changes in brain activity

**Figure 4:**
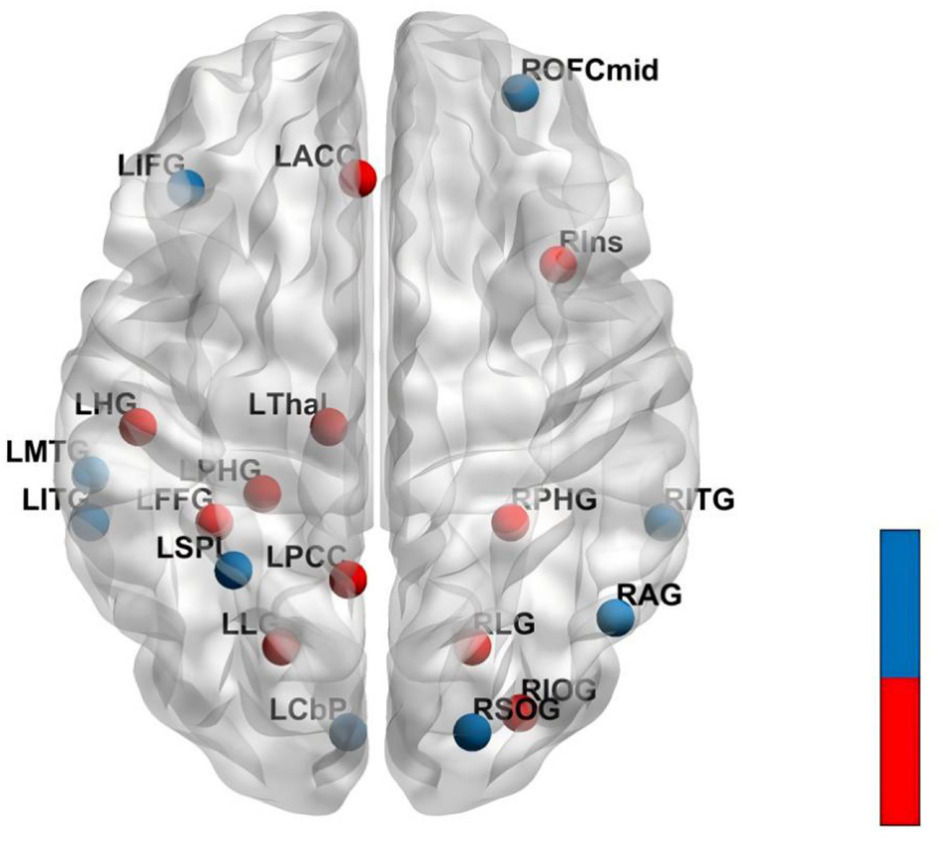
Brain regions changes induced by acupuncture. The figure shows a dorsal view of the brain. Red spheres = increased activity; Blue spheres = decreased activity. Increased Activity (Red): LPHG, Left Parahippocampal Gyrus; LPCC, Left Posterior Cingulate Cortex; LFFG, Left Fusiform Gyrus; LHG, Left Heschl's Gyrus; LLG, Left Lingual Gyrus; RLG, Right Lingual Gyrus; RIOG, Right Inferior Occipital Gyrus; RPHG, Right Parahippocampal Gyrus; LThal, Left Thalamus; RIns, Right Insula; LACC, Left Anterior Cingulate Cortex. Decreased Activity (Blue): RAG, Right Angular Gyrus; ROFCmid, Right Orbital Middle Frontal Gyrus; RSOG, Right Superior Occipital Gyrus; LCbP, Left Cerebellum Posterior Lobe; LITG, Left Inferior Temporal Gyrus; RITG, Right Inferior Temporal Gyrus; LIFG, Left Inferior Frontal Gyrus; LMTG, Left Middle Temporal Gyrus; LSPL, Left Superior Parietal Lobule.

Five rs-fMRI studies evaluated acupuncture's effects on brain functional activity in PISCI (Figure 4). Acupuncture-induced brain changes primarily involved regions associated with memory, executive function, visuospatial processing, and attention.

###### 3.8.2.1.1 Memory-related regions

Enhanced activity was observed in memory structures across multiple studies. The para hippocampal gyrus showed increased activity in 3/5 studies (60%), with elevated ALFF in the right para hippocampal gyru and increased DC and local efficiency in the left para hippocampal gyrus. Hippocampal frontal connectivity was strengthened bilaterally, with enhanced connections between the left hippocampus and right middle/inferior frontal gyri, and between the right hippocampus and left frontal regions and parietal lobe. The left thalamus also showed increased ALFF.

###### 3.8.2.1.2 Executive function-related regions

Executive networks showed mixed modulation patterns. Hippocampal-frontal connectivity was enhanced bilaterally, and the left anterior cingulate gyrus exhibited increased ALFF. The left posterior cingulate gyrus showed increased DC. However, decreased activity occurred in the left inferior frontal gyrus (reduced ALFF) and right orbitofrontal cortex (decreased DC).

###### 3.8.2.1.3 Visuospatial processing regions

The fusiform gyrus, critical for visual object recognition, showed consistent increases across 3/5 studies (60%), with elevated fALFF, increased DC and increased local efficiency. The lingual gyrus also demonstrated enhanced activity in 3/5 studies (60%), with increased DC and node centrality bilaterally. Occipital regions showed mixed patterns: the left inferior occipital gyrus showed increased DC and node centrality but decreased ALFF, the right inferior occipital gyrus showed increased local efficiency, and the right superior occipital gyrus exhibited decreased fALFF.

###### 3.8.2.1.4 Attention-related regions

The cingulate cortex showed increased activity in 2/5 studies (40%): left anterior cingulate gyrus and left posterior cingulate gyrus. The right insula demonstrated increased ALFF. Conversely, the right angular gyrus showed decreased DC and local efficiency, and the left superior parietal lobule showed reduced ALFF.

###### 3.8.2.1.5 Other regions

The left transverse temporal gyrus showed increased DC and local efficiency. Decreased activity was observed in bilateral inferior temporal gyri, left middle temporal gyrus, and left posterior cerebellar lobe.

##### 3.8.2.2 Acupuncture-induced changes in brain metabolism

Three studies (Su, 2016; Wang et al., 2014; Zhang et al., 2020) used MRS to assess metabolite ratios in regions including the infarct area, hippocampus, and frontal white matter. Among the three studies that employed MRS, all observed significant increases in NAA/Cr after acupuncture. Two of these three studies also reported reductions in MI/Cr, and two observed decreases in Cho/Cr.

#### 3.8.3 Correlation between neuroimaging and clinical outcomes

Three studies (3/8, 37.5%) examined associations between acupuncture-related brain changes and cognitive test scores. Two studies found a negative correlation between the area under the curve (AUC) of DC in the left inferior occipital gyrus and MoCA scores after acupuncture. One study did not identify any significant correlations between fALFF, ReHo, or DC values in altered regions and neuropsychological scores.

#### 3.8.4 Potential brain network changes induced by acupuncture

**Figure 5:**
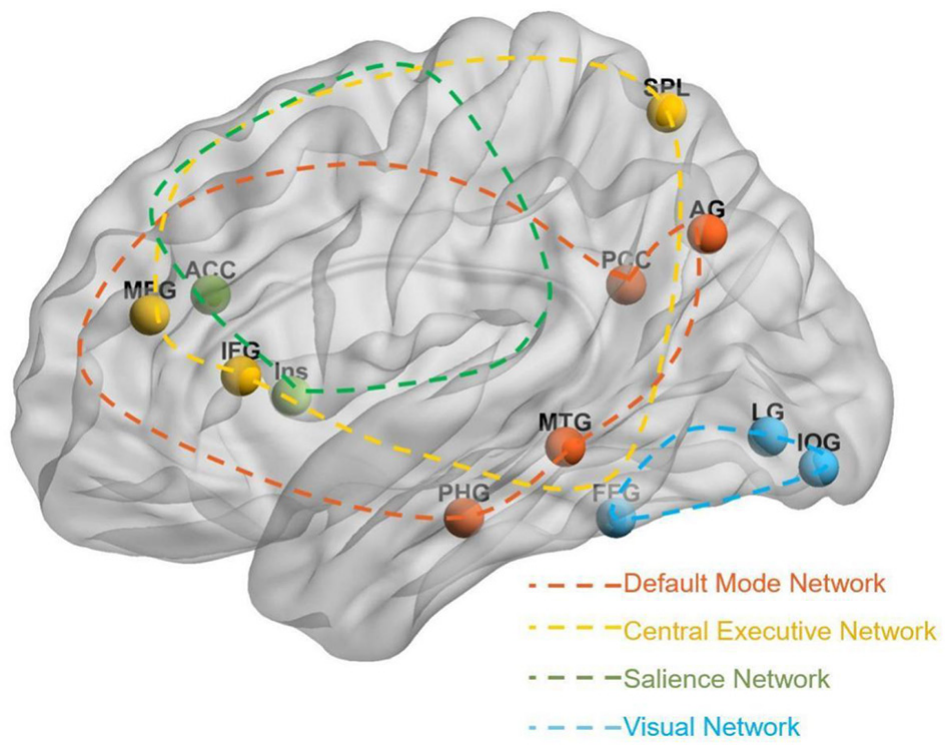
Brain networks potentially induced by acupuncture. Lateral view (left hemisphere) showing four major functional networks potentially modulated by acupuncture treatment. Default Mode Network (DMN, orange): PHG, Parahippocampal Gyrus; PCC, Posterior Cingulate Cortex; MTG, Middle Temporal Gyrus; AG, Angular Gyrus. Central Executive Network (CEN, yellow): MFG, Middle Frontal Gyrus; IFG, Inferior Frontal Gyrus; SPL, Superior Parietal Lobule. Salience Network (SN, green): Ins, Insula; ACC, Anterior Cingulate Cortex. Visual Network (VN, blue): LG, Lingual Gyrus; IOG, Inferior Occipital Gyrus; FFG, Fusiform Gyrus.

**Figure 6:**
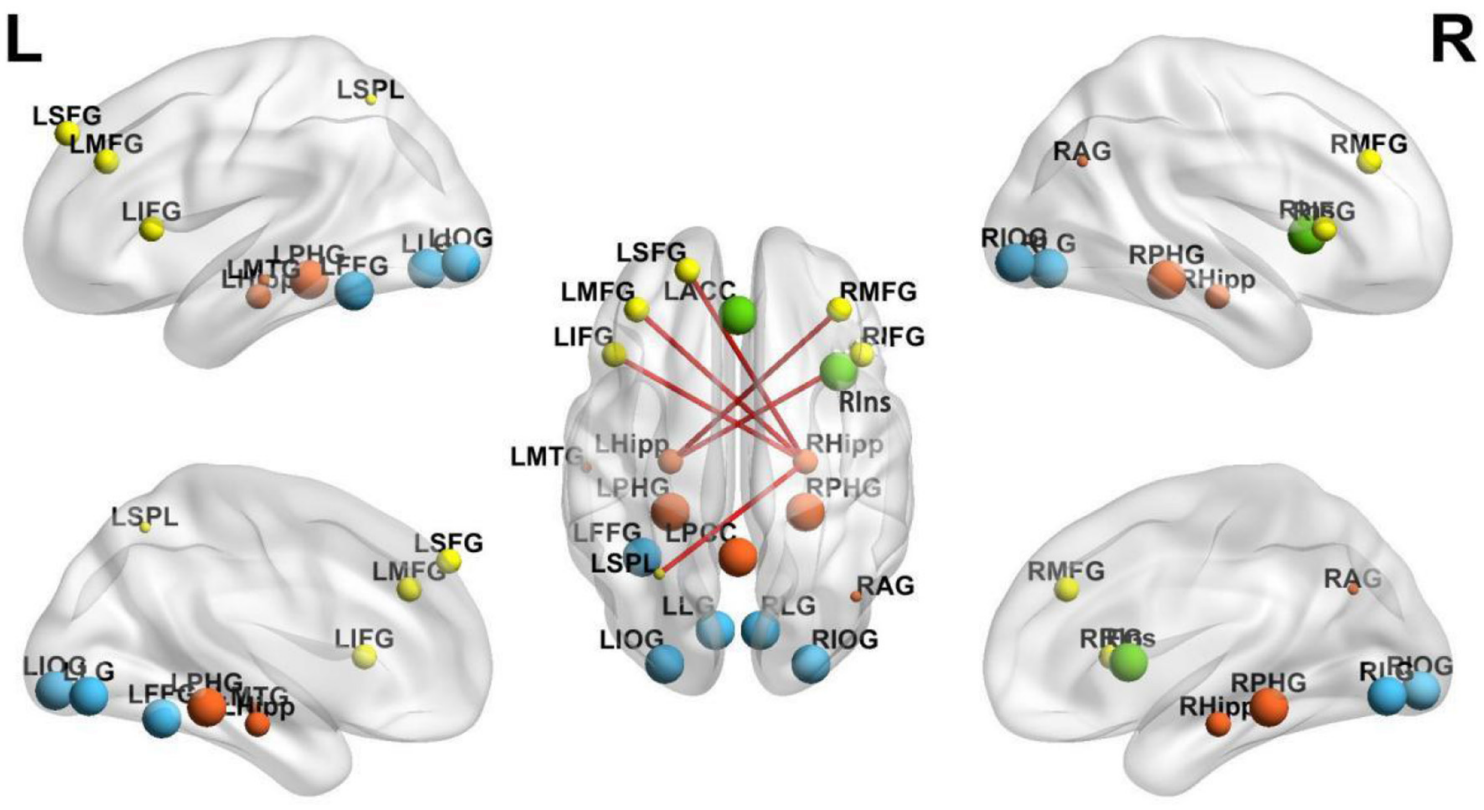
Brain network alterations in regional activity and functional connectivity after acupuncture. The figure shows brain activity changes and functional connectivity alterations across four views (left lateral, right lateral, left medial, right medial). Colored spheres represent network nodes with activity changes; red lines indicate significantly enhanced functional connectivity between regions. Sphere size encoding: Smallest spheres = decreased activity; Largest spheres = increased activity; Medium-sized spheres = regions showing functional connectivity changes only (without significant local activity changes). Network color coding: Orange = DMN, Yellow = CEN, Green = SN, Blue = VN. DMN (orange): LHipp, left hippocampus; RHipp, right hippocampus; LPHG, left parahippocampal gyrus; RPHG, right parahippocampal gyrus; LPCC, left posterior cingulate cortex; RAG, right angular gyrus; CEN (yellow): LSFG, left superior frontal gyrus; LMFG, left middle frontal gyrus; RMFG, right middle frontal gyrus; LIFG, left inferior frontal gyrus; RIFG, right inferior frontal gyrus; LSPL, left superior parietal lobule; SN (green): LACC, left anterior cingulate cortex; RIns, right Insula; VN (blue): LFFG, left fusiform gyrus; LLG, left lingual gyrus; RLG, right lingual gyrus; LIOG, left inferior occipital gyrus; RIOG, right inferior occipital gyrus.

Affected regions mapped onto several functional networks (Figure 5): the default mode network (DMN; para hippocampal gyrus, posterior cingulate gyrus, middle temporal gyrus, angular gyrus); the central executive network (CEN; orbitofrontal cortex, inferior frontal gyrus, superior parietal lobule); the salience network (SN; insula, anterior cingulate gyrus); and the visual network (VN; lingual gyrus, inferior occipital gyrus, fusiform gyrus). Figure 6 illustrates the modulation of brain regions within brain networks and associated changes in FC.

#### 3.8.5 Correlation between neuroimaging findings and detailed acupuncture

None of the included studies directly analyzed associations between specific acupuncture parameters and neuroimaging outcomes; however, a descriptive analysis can be conducted based on STRICTA details and study results.

Among the five rs-fMRI studies, most employed prescriptions centered on GV20 (4/5, 80%) and EX-HN1 (3/5, 60%), often combined with PC6 (3/5, 60%). Only one study used scalp acupuncture (1/5, 20%). In these studies focusing on vertex acupoints, neuroimaging results demonstrated increased activity in the left fusiform gyrus (3/5, 60%), para hippocampal gyrus (3/5, 60%), cingulate gyrus (2/5, 40%), and bilateral lingual gyri (2/5, 40%), alongside decreased activity in the right angular gyrus (2/5, 40%). Regarding techniques, four studies used manual acupuncture (4/5, 80%) and one used electro acupuncture (1/5, 20%). The electro acupuncture study reported reduced ALFF in the left inferior occipital gyrus, whereas two manual acupuncture studies showed increased DC and node centrality in the same region.

Three additional studies used MRS with target regions including the area of cerebral infarction, bilateral frontal lobe white matter, and the hippocampal region. Frequently selected acupoints included LI4 (3/3, 100%), LR3 (3/3, 100%), GV24 (2/3, 66.7%), EX-HN1 (2/3, 66.7%), and GV20 (2/3, 66.7%); all used manual acupuncture. MRS findings consistently demonstrated increased NAA/Cr ratios (3/3, 100%), together with decreased Cho/Cr (3/3, 100%) and MI/Cr (2/3, 66.7%).

## 4 Discussion

This SR evaluated neuroimaging studies on acupuncture for PISCI in accordance with PRISMA. Eight studies (*n* = 397) were included. The primary objective was to summarize neuroimaging evidence on the effects of acupuncture in PISCI and to explore potential neural mechanisms.

### 4.1 Characteristics of included neuroimaging studies

All included studies were conducted in China, reflecting the widespread clinical use of acupuncture in that context. However, the lack of studies from other countries may limit generalizability to populations with different cultural backgrounds and acceptance of acupuncture. The included studies comprised five RCTs and three non-RCTs. Quality assessment revealed that two RCTs were at high risk of bias, primarily due to inadequately reported randomization process and lack of allocation concealment; the remaining three were judged to have “some concerns,” largely related to deviations from intended interventions and incomplete outcome reporting. These methodological weaknesses represent critical threats to internal validity and indicate that findings should be interpreted with caution. Future research should adhere closely to methodological standards outlined in the Cochrane Handbook for Systematic Reviews of Interventions to improve rigor and transparency, thereby enhancing the overall credibility of the evidence.

All included studies had small sample sizes (fewer than 80 participants), which fundamentally limits statistical power and reproducibility. Prior research has shown that (Klapwijk et al., 2021) small neuroimaging samples may overestimate effect sizes and reduce reproducibility. Without predefined analysis plans, especially for ROI analyses, the risk of false positives further increases (Gentili et al., 2021). Cross-validation may mitigate this risk but cannot fully compensate for inadequate sample sizes (Goltermann et al., 2023). Additionally, all studies recruited participants from tertiary care centers, potentially limiting generalizability to community settings. Future studies should increase sample sizes and standardize data-analysis pipelines. Regarding participant characteristics, seven of the eight studies included more men than women, consistent with the higher global incidence of ischemic stroke in males, especially under the age of 75 (de Miguel-Yanes et al., 2021). Sex may influence neural repair and cognitive recovery after stroke (Madsen and Guo, 2020; Zinman et al., 2023); thus, future trials should explicitly include sex as a biological variable.

Cross-study replication of findings strengthens the credibility of the evidence. Among the five rs-fMRI studies, several brain regions showed consistent changes: the para hippocampal gyrus, fusiform gyrus, and lingual gyrus demonstrated enhanced activity across independent different studies. In contrast, changes in other regions were reported in single studies only, representing preliminary observations requiring validation. However, due to the limited number of included studies and methodological heterogeneity, quantitative meta-analysis was not feasible, which constrained our ability to estimate pooled effect sizes and assess publication bias. Notably, the predominance of positive findings among small-sample studies suggests potential publication bias, although formal statistical testing was not possible due to insufficient study numbers.

The included studies varied widely in acupuncture methods, acupoint selections, and treatment protocols. Seven studies used manual acupuncture, one used electro acupuncture, and only five reported practitioner qualifications. Variability in techniques, needle retention time, and stimulation intensity could influence treatment effects and reproducibility (Yang et al., 2013). Several studies did not fully comply with STRICTA, with incomplete reporting of details such as deqi sensations or acupoint rationale; only five studies described patient responses to acupuncture. Evidence suggests that (Li et al., 2015a; Shi et al., 2016) deqi is associated with activation of regions such as the insula and cingulate gyrus, potentially underlying central effects of acupuncture. Standardization and consistent adherence to STRICTA are therefore critical for improving comparability and reliability.

Regarding acupoint selection, GV20 and EX-HN1 were most frequently used; both are located at the vertex. Unlike distal points commonly used for Alzheimer's disease (AD) or mild cognitive impairment (MCI) (Yin et al., 2023a,b), these acupoints directly stimulate cortical regions beneath the skull. Neuroimaging studies indicate that (Deng et al., 2015; Kong et al., 2024; Wei et al., 2021) these regions correspond to prefrontal and parietal cortices—areas related to executive function, attention, and working memory. Animal research further suggests that (Zheng et al., 2020) GV20 may promote neuroplasticity and neurotrophic factor expression, potentially improving cognitive function.

For outcome measurement, the most commonly used cognitive assessment tools were MoCA and MMSE. Numerous studies indicate that MoCA is more sensitive than MMSE for detecting MCI, making it suitable for identifying early post-stroke cognitive deficits (Dong et al., 2014; Lestari et al., 2017; Pendlebury et al., 2012). MMSE is more commonly used for dementia screening and for assessing overall cognitive function (Kawada, 2018; Lyrakos et al., 2014). Additional tools included WMS-RC, TMT, AVLT, and DS. WMS-RC is widely used in China for assessing verbal and visual memory (Ding et al., 2009). TMT is sensitive to attentional and executive dysfunction and, when combined with MoCA, can improve screening accuracy for post-stroke vascular cognitive impairment (VCI) (Dong et al., 2014; Kodama et al., 2022). AVLT assesses auditory-verbal memory (Xin-Xian, 2012), while the DS evaluates working memory and correlates with daily functional status (Fitri et al., 2020; Leung et al., 2011). Overall, although MoCA and MMSE remain the primary screening tools in PISCI, they have limitations for domain-specific deficits. Future studies should incorporate more targeted assessments (e.g., AVLT, DS, TMT) and consider emerging approaches such as digital cognitive tools (Bateh et al., 2025) or virtual reality-integrated cognitive tasks (Gunawan et al., 2024) to enhance comprehensiveness and sensitivity.

### 4.2 Findings of acupuncture treatment on neuroimaging of PISCI

In this SR, rs-fMRI and MRS were used to investigate the effects of acupuncture on brain functional activity and neurometabolic levels. It should be noted that all included studies reported within-group pre-post comparisons as their primary outcome. Two studies included healthy controls as normative references, but the primary analysis in all studies focused on within-group pre-post changes in acupuncture-treated PISCI patients. Therefore, the findings below reflect treatment-associated changes from pre- to post-treatment. However, studies varied in design (some lacked parallel control groups while others were randomized controlled trials), which should be considered when interpreting causal implications.

Rs-fMRI, a non-invasive BOLD-based technique, characterizes regional neural activity and large-scale network organization during rest (Zhen et al., 2018) and has been widely applied in studies of cognitive impairment and post-stroke brain functional reorganization (Cha et al., 2015; Golestani et al., 2013). Common rs-fMRI metrics include ALFF/fALFF (amplitude of spontaneous activity) (Wang et al., 2023), ReHo (local synchronization) (Han et al., 2018), and FC (interregional coupling) (Hua et al., 2024). FC analyses are often combined with graph theory to extract the topological features of brain networks. Frequently used graph-based indicators include DC and local efficiency. DC refers to the number of connections a given brain region has with other regions, indicating its hub-like role within the network (Kuhnert et al., 2012). Local efficiency measures the efficiency of information transmission among neighboring nodes, reflecting the integrative capacity of local subnetworks (Sang et al., 2020). Integrating these multilevel metrics provides a comprehensive framework for probing central regulatory mechanisms of acupuncture in PISCI.

MRS, a non-invasive metabolic technique, quantifies relative concentrations of brain metabolites (e.g., NAA, Cho, and MI) to assess neuronal function, glial activity, and inflammation (Tumati et al., 2013). The included studies demonstrated that acupuncture significantly increased NAA/Cr in the hippocampus and prefrontal cortex and reduced MI/Cr, suggesting improved neuronal function and attenuated inflammatory responses. One study reported a positive correlation between NAA/Cr and cognitive scores, supporting its potential as a neuroimaging biomarker for PISCI (Meng et al., 2016). It should be noted that MRS-derived metabolite ratios may vary depending on both the anatomical region examined and the post-stroke recovery phase. Previous studies included in this review measured metabolite levels from different brain regions, such as the hippocampus, frontal white matter, or peri-infarct cortex, and at various stages of stroke recovery (acute, subacute, or chronic). These regional and temporal differences can independently influence NAA, Cho, and MI concentrations, reflecting distinct metabolic and reparative processes at different stages of neural reorganization. Therefore, while the current synthesis demonstrates a generally consistent pattern, these results should be interpreted as reflecting an overall trend toward metabolic improvement rather than a precise quantitative convergence. Future studies with standardized voxel placement, acquisition parameters, and longitudinal follow-up are needed to clarify how acupuncture modulates regional and temporal metabolic dynamics after stroke.

Notably, most included studies used single-modality imaging, with limited multimodal integration, which may constrain comprehensive mechanistic understanding. Different techniques offer complementary strengths: DTI reflects post-stroke white matter microstructural damage and remodeling (Auriat et al., 2015); fNIRS, with high temporal resolution and portability, enables real-time prefrontal hemodynamic monitoring during acupuncture (Annavarapu et al., 2018); and Electroencephalogram and Magnetoencephalography (EEG/MEG) capture rapid, acupuncture-related electrophysiological activity (Hall et al., 2014). Future research should adopt multimodal strategies to elucidate acupuncture-mediated central mechanisms and provide robust evidence for precise interventions.

The included studies focused on the sustained effects of acupuncture in PISCI patients. One study revealed that acupuncture significantly enhanced hippocampus-prefrontal functional connectivity (including the middle, inferior, and superior frontal gyri). Consistent with this, a multimodal MRI study (Hosseini et al., 2016) identified hippocampal structural damage as closely associated with cognitive dysfunction and as a major predictor of post-stroke dementia. The hippocampus plays a central role in PSCI, with functional damage strongly linked to declines in memory and learning capacity (Delattre et al., 2017; Kliper et al., 2016). The prefrontal cortex supports executive function, attention, and memory, and reduced connectivity in this region has been associated with cognitive decline (Li et al., 2015b). By enhancing connectivity within this pathway, acupuncture may promote neuroplasticity and network reorganization, thereby improving cognitive function after stroke (Ding et al., 2014; Yang et al., 2014).

This review also indicates that acupuncture modulates several cognition-related regions, including the para hippocampal gyrus, fusiform gyrus, inferior occipital gyrus, cingulate gyrus, transverse temporal gyrus, lingual gyrus, and angular gyrus. The para hippocampal gyrus links the hippocampus with other cortical areas and is essential for memory encoding and spatial navigation. Studies have shown that both the hippocampus and para hippocampal gyrus often show concomitant abnormalities in PISCI and AD, which relate to memory impairment (Jung et al., 2020; Lu et al., 2024). The fusiform gyrus, implicated in visual recognition, exhibits abnormalities in PISCI (Cheng et al., 2021), and serves as an early biomarker in AD/MCI (Cai et al., 2015; Whitwell et al., 2007). The lingual and inferior occipital gyri, components of the visual network, may affect visuospatial abilities and information processing after stroke (Smagula et al., 2018). The cingulate gyrus is a hub for attention and executive control and has been associated with impaired cognitive regulation post-stroke (Lang et al., 2023). The transverse temporal gyrus, primarily involved in auditory processing, may signal language-related cognitive impairment when disrupted, particularly in left-hemisphere stroke (Delattre et al., 2017). The angular gyrus contributes to semantic processing, attentional regulation, and working memory; decreased functional connectivity of this region has been linked to lower MoCA scores in PISCI (Jiang et al., 2014). Collectively, these regions support multiple cognitive domains, including memory, executive function, attentional control, and visual perception. Dysfunction of these regions is closely associated with PISCI. The present findings suggest that acupuncture may modulate these regions and support network-level reorganization. Future work should clarify the roles of these brain areas across cognitive domains and identify the principal pathways and targets through which acupuncture exerts its effects.

Acupuncture may also modulate several functional brain networks implicated in PISCI, including the DMN, CEN, SN, and VN. The DMN, associated with high-level cognitive processes, often shows reduced connectivity after stroke, correlating with cognitive decline (Yue et al., 2023). The CEN, responsible for executive control and working memory, typically shows hypo-activation post-stroke; acupuncture may help modulate this reduction (Hoffmann, 2020). The SN functions as a regulatory switch between the DMN and CEN and frequently demonstrates impaired efficiency in PISCI; acupuncture may help restore this modulatory role (Wang et al., 2024a). The VN, which supports visual information processing, can also be disrupted after a stroke; acupuncture may aid the recovery of visual perception and related functions (Liu et al., 2017). Recent neuroimaging studies increasingly highlight abnormalities in both static and dynamic connectivity within and between the DMN, CEN, and SN in stroke-related cognitive deficits (Veldsman et al., 2020; Wang et al., 2024b; Yue et al., 2023). These findings suggest that acupuncture may support cognitive recovery by modulating network connectivity, particularly in attention, memory, and executive control. Further research is needed to clarify how acupuncture influences network interactions and contributes to reorganization after stroke.

However, it is important to note that most included studies employed rs-fMRI to examine spontaneous brain activity and functional connectivity, without task-based validation to directly link observed neural changes to specific cognitive processes. As a result, the functional interpretation of regional activation changes remains correlational and inferential. For example, while altered activity in the fusiform or lingual gyrus may be associated with visual processing, without concurrent cognitive task performance, we cannot definitively establish whether these neural changes translate into measurable improvements in visuospatial function. Future studies should incorporate task-based fMRI paradigms that probe memory, attention, executive function, and other cognitive domains to validate the functional relevance of acupuncture-induced neural modulation.

### 4.3 Potential factors influencing the neural effects of acupuncture

Although this SR provides a preliminary synthesis of neuroimaging evidence on acupuncture for PISCI, it is important to note that multiple factors may modulate acupuncture-induced brain functional changes.

Intervention-related parameters represent a key set of determinants. Evidence suggests that timing may critically influence neuroplasticity and functional recovery. One study reported (Li et al., 2024) that treatment initiated during the acute stage of stroke was associated with a lower risk of disability at 6 months, and another neuroimaging study (Wang et al., 2022b) showed stronger neural responses when acupuncture was applied within the 1^st^ month after stroke, indicating a time-dependent effect. Moreover, differences in treatment dose, such as duration, frequency, and stimulation intensity, also play an essential role. An fMRI study (Xiang et al., 2021) revealed frequency-dependent alterations in BOLD oscillations linked to analgesia, and an animal experiment demonstrated (Wang et al., 2024b) that moderate-intensity stimulation produced optimal analgesic and immune regulatory effects. Acupoint selection likewise contributes to variability. Although GV20 and EX-HN1 were commonly selected in included studies, different acupoints or combinations can elicit distinct activation patterns. Studies have indicated that (Yuan et al., 2021) stimulation of language-related acupoints induced different cortical activations, and in hypertension (Zhang et al., 2019), combined LR3+KI3 stimulation activated broader frontal, insular, and parietal regions than single-point interventions.

In addition, stroke-related clinical characteristics significantly affect the therapeutic effects of acupuncture. In acute ischemic stroke (Tsai et al., 2024), greater neurological impairment limited the extent of functional recovery achieved with acupuncture, although anti-inflammatory benefits were still observed. Lesion location is a key factor influencing acupuncture effects. A study of post-stroke dysphagia suggested that (Qiu et al., 2021) efficacy depended on lesion distribution in the brainstem and cortico-subcortical extension region, and another study reported (Zhang et al., 2024) distinct acupuncture-induced activation patterns across infarct locations. Furthermore, ischemia duration determines the degree of neuronal necrosis and the therapeutic window. An animal experiment demonstrated that (Guo et al., 2025) acupuncture administered within 24 h after ischemia significantly reduced infarct volume and improved neurological scores, whereas delayed intervention produced weaker effects.

Overall, the included studies did not sufficiently address these factors, limiting understanding of neural effects. Future research should incorporate intervention parameters and stroke characteristics into study design and adhere to STRICTA and the Consolidated Standards of Reporting Trials (CONSORT) guidelines to ensure transparency and scientific rigor.

### 4.4 Comparison with other studies

A study of neuroimaging in AD (Yin et al., 2023a) reported acupuncture-induced activation in the hippocampus, prefrontal cortex, and parietal regions, along with enhanced integration of the DMN, CEN, and fronto parietal network (FPN). Another review focused on MCI (Yin et al., 2023b) highlighted modulation of the anterior cingulate cortex and insula, involving the DMN and SN. An fMRI meta-analysis (Ma et al., 2022) further revealed increased activation in the thalamic, frontal, and cingulate regions in MCI after acupuncture, suggesting potential benefits for attention and executive function. While broadly consistent, these studies differ in focus. The present review targets PISCI and indicates modulation of visual-related brain regions (lingual gyrus, inferior occipital gyrus, fusiform gyrus), which are less reported in AD or MCI. This discrepancy may reflect the heterogeneity of stroke lesions, which can affect motor, language, and visual systems in addition to memory, complicating cognitive assessment (Wang, 2020). Thus, cognitive impairment after stroke may involve multimodal sensory and attentional deficits beyond memory decline. These findings contribute to a more comprehensive understanding of the neural mechanisms underlying acupuncture in PISCI and provide a comparative framework for evaluating differential effects across cognitive disorders. Future research should include cross-condition comparisons, multimodal imaging, and task-based designs to better characterize the neural substrates of acupuncture.

### 4.5 Limitations

This review has several limitations. First, most included studies had small sample sizes and heterogeneous designs, potentially affecting the generalizability. Second, key factors such as stroke type, lesion location, recovery phase, concurrent rehabilitation programs, and pharmacotherapy were rarely reported or controlled across studies, which may influence brain reorganization and intervention efficacy. Without systematic control of these variables, the specific contribution of acupuncture may be overstated. Future studies should employ stricter protocols for documenting and adjusting for these clinical factors. Third, variability in imaging protocols, analysis methods, and outcome measures precluded meta-analysis or quantitative integration.

Fourth, only eight studies were included in the final analysis, and several were dissertations, which may limit the representativeness and robustness. Fifth, formal assessment of publication bias was not feasible due to the small number of studies and the absence of pooled quantitative data; nevertheless, the predominance of positive findings suggests that potential publication bias cannot be ruled out. Finally, all included studies were conducted in China, which may limit the generalizability due to potential cultural and methodological factors. Cultural factors such as patient expectations and beliefs about acupuncture, which vary across populations, may influence placebo responses and neural patterns. Acupuncture practice varies internationally in practitioner training, technique standardization, and acupoint selection, potentially affecting neural responses. Cross-country differences in neuroimaging protocols and population characteristics (e.g., stroke etiology, genetic backgrounds) may also impact comparability. While the consistency across multiple independent Chinese studies supports reliability, international multicenter studies with standardized protocols are needed to validate whether these mechanisms generalize across diverse populations and cultural contexts.

## 5 Conclusions

This review provides preliminary neuroimaging evidence supporting the potential benefits of acupuncture for PISCI. The findings suggest that the possible mechanisms of acupuncture for PISCI involve changes in the activity and enhanced functional connectivity of cognition-related brain regions. Additionally, acupuncture may influence brain networks and regulate neurochemical metabolites within cognition-related regions. However, as this field remains in its early stages, further validation is needed. Future studies should focus on well-designed, multicenter randomized controlled trials (RCTs) with large sample sizes and incorporate multiple neuroimaging techniques to better clarify and verify the neural mechanisms of acupuncture in PISCI.

## Data Availability

The original contributions presented in the study are included in the article/Supplementary material, further inquiries can be directed to the corresponding author.

## References

[B1] Annavarapu R. N. Kathi S. Vadla V. K. (2018). Non-invasive imaging modalities to study neurodegenerative diseases of aging brain. J. Chem. Neuroanat. 95, 54–69. doi: 10.1016/j.jchemneu.2018.02.006 29474853

[B2] Auriat A. Neva J. Peters S. Ferris J. Boyd L. (2015). A review of transcranial magnetic stimulation and multimodal neuroimaging to characterize post-stroke neuroplasticity. Front. Neurol. 6:00226. doi: 10.3389/fneur.2015.00226 26579069 PMC4625082

[B3] Bateh K. Saurman J. Bartsch B. Xu Y. Aboul-Nour H. Hanson A. . (2025). Abstract tp45: advancements in digital cognitive assessments for post-stroke patients: a scoping review. Stroke 56, (Suppl 1). doi: 10.1161/str.56.suppl_1.TP45

[B4] Benedict R. H. B. Amato M. P. DeLuca J. Geurts J. J. G. (2020). Cognitive impairment in multiple sclerosis: clinical management, mri, and therapeutic avenues. Lancet Neurol. 19, 860–871. doi: 10.1016/S1474-4422(20)30277-5 32949546 PMC10011205

[B5] Beristain X. Golombievski E. (2015). Pharmacotherapy to enhance cognitive and motor recovery following stroke. Drugs Aging 32, 765–772. doi: 10.1007/s40266-015-0299-0 26423272

[B6] Bournonville C. Hénon H. Dondaine T. Delmaire C. Bombois S. Mendyk A. . (2018). Identification of a specific functional network altered in poststroke cognitive impairment. Neurology 90, e1879–e1888. doi: 10.1212/WNL.0000000000005553 29678937

[B7] Cai S. Chong T. Zhang Y. Li J. Von Deneen K. Ren J. . (2015). Altered functional connectivity of fusiform gyrus in subjects with amnestic mild cognitive impairment: a resting-state fmri study. Front. Hum. Neurosci. 9:00471. doi: 10.3389/fnhum.2015.00471 26379534 PMC4550786

[B8] Cha J. Hwang J. Jo H. Seo S. Na D. Lee J. (2015). Assessment of functional characteristics of amnestic mild cognitive impairment and alzheimer's disease using various methods of resting-state fmri analysis. Biomed. Res. Int. 2015:907464. doi: 10.1155/2015/907464 26180816 PMC4477185

[B9] Chavez L. M. Huang S. Macdonald I. Lin J. Lee Y. Chen Y. (2017). Mechanisms of acupuncture therapy in ischemic stroke rehabilitation: a literature review of basic studies. Int. J. Mol. Sci. 18:ijms18112270. doi: 10.3390/ijms18112270 29143805 PMC5713240

[B10] Cheng R. Chen L. Liu X. Luo T. Gong J. Jiang P. (2021). Changes in gray matter asymmetries of the fusiform and parahippocampal gyruses in patients with subcortical ischemic vascular disease. Front. Neurol. 11:603977. doi: 10.3389/fneur.2020.603977 33551966 PMC7859431

[B11] Cicerone K. D. Langenbahn D. M. Braden C. Malec J. F. Kalmar K. Fraas M. . (2011). Evidence-based cognitive rehabilitation: updated review of the literature from 2003 through 2008. Arch. Phys. Med. Rehabil. 92, 519–530. doi: 10.1016/j.apmr.2010.11.015 21440699

[B12] de Miguel-Yanes J. M. Jiménez-García R. López-De-Andrés A. Hernández-Barrera V. de Miguel-Díez J. Méndez-Bailón M. . (2021). The influence of sex on ischemic stroke incidence, therapeutic procedures and in-hospital mortality: results of the spanish national hospital discharge. Int. J. Clin. Pract. 75:e14984. doi: 10.1111/ijcp.14984 34637167

[B13] Delattre C. Bournonville C. Auger F. Lopes R. Delmaire C. Hénon H. . (2017). Hippocampal deformations and entorhinal cortex atrophy as an anatomical signature of long-term cognitive impairment: from the mcao rat model to the stroke patient. Transl. Stroke Res. 9, 294–305. doi: 10.1007/s12975-017-0576-9 29034421

[B14] Deng D. Duan G. Liu Y. Liao H. Wang G. Liu H. . (2015). Altered amplitude of low-frequency fluctuation induced by acupuncture at baihui acupoint: a pilot functional mri study. Alternat. Integr. Med. 2015:100197. doi: 10.4172/2327-5162.1000197

[B15] Ding S. Gao B. Cai M. Li Y. Sheng L. (2009). Application analysis of Wechsler Memory Scale and Clinical Memory Scale in disability identification after craniocerebral injury. Chinese J. Clin. Psychol. 17, 457–458. doi: 10.16128/j.cnki.1005-3611.2009.04.033

[B16] Ding X. Li C. Y. Wang Q. S. Du F. Ke Z. Peng F. . (2014). Patterns in default-mode network connectivity for determining outcomes in cognitive function in acute stroke patients. Neuroscience 277, 637–646. doi: 10.1016/j.neuroscience.2014.07.060 25090922

[B17] Dong Y. Slavin M. Chan B. Venketasubramanian N. Sharma V. Collinson . (2014). Improving screening for vascular cognitive impairment at three to six months after mild ischemic stroke and transient ischemic attack. Int. Psychogeriatr. 26, 787–793. doi: 10.1017/S1041610213002457 24423626

[B18] Du S. Wang X. Zhu W. Ye Y. Yang J. Ma S. . (2018). Acupuncture inhibits txnip-associated oxidative stress and inflammation to attenuate cognitive impairment in vascular dementia rats. CNS Neurosci. Ther. 24, 39–46. doi: 10.1111/cns.12773 29110407 PMC6489958

[B19] Eshaghi Ghalibaf M. H. Rajabian A. Parviz M. Akbarian M. Amirahmadi S. Vafaee F. . (2023). Minocycline alleviated scopolamine-induced amnesia by regulating antioxidant and cholinergic function. Heliyon 9:e13452. doi: 10.1016/j.heliyon.2023.e13452 36816250 PMC9929315

[B20] Ferrari A. J. Santomauro D. F. Aali A. Abate Y. H. Abbafati C. Abbastabar H. . (2024). Global incidence, prevalence, years lived with disability (ylds), disability-adjusted life-years (dalys), and healthy life expectancy (hale) for 371 diseases and injuries in 204 countries and territories and 811 subnational locations, 1990–2021: a systematic analysis for the global burden of disease study 2021. Lancet 403, 2133–2161. doi: 10.1016/S0140-6736(24)00757-8 38642570 PMC11122111

[B21] Fitri F. I. Fithrie A. Rambe A. (2020). Association between working memory impairment and activities of daily living in post-stroke patients. Medicinski glasnik: official publication of the Medical Association of Zenica-Doboj Canton, Bosnia and Herzegovina. Med. Glas. 17:2. doi: 10.17392/1135-20 32489085

[B22] Gentili C. Cecchetti L. Handjaras G. Lettieri G. Cristea I. A. (2021). The case for preregistering all region of interest (roi) analyses in neuroimaging research. Eur. J. Neurosci. 53, 357–361. doi: 10.1111/ejn.14954 32852863

[B23] Golestani A. Tymchuk S. Demchuk A. Goodyear B. (2013). Longitudinal evaluation of resting-state fmri after acute stroke with hemiparesis. Neurorehabil. Neural Repair 27, 153–163. doi: 10.1177/1545968312457827 22995440

[B24] Goltermann J. Winter N. R. Gruber M. Fisch L. Richter M. Grotegerd D. . (2023). Cross-validation for the estimation of effect size generalizability in mass-univariate brain-wide association studies. PsyAxiv preprint. doi: 10.1101/2023.03.29.534696

[B25] Gunawan H. Gunawan I. Hambarsari Y. Danuaji R. Hamidi B. Benedictus B. (2024). Virtual reality intervention for improving cognitive function in post-stroke patient: a systematic review and meta-analysis. PsyAxiv preprint. doi: 10.2139/ssrn.5295244

[B26] Guo L. Huang Z. Huang L. Liang J. Wang P. Zhao L. . (2021). Surface-modified engineered exosomes attenuated cerebral ischemia/reperfusion injury by targeting the delivery of quercetin towards impaired neurons. J. Nanobiotechnol. 19:141. doi: 10.1186/s12951-021-00879-4 34001136 PMC8130330

[B27] Guo Y. Hu S. Luo S. Tu L. Tang Y. Zeng F. (2025). Experimental evidence-based construction of electroacupuncture for ischemic stroke: a meta-analysis and systematic review. Front. Neurol. 16:1491132. doi: 10.3389/fneur.2025.1491132 39974363 PMC11835673

[B28] Hall E. Robson S. Morris P. Brookes M. (2014). The relationship between meg and fmri. NeuroImage 102, 80–91. doi: 10.1016/j.neuroimage.2013.11.005 24239589

[B29] Han J. Yang Y. Wang Y. Feng J. Song C. Wu W. . (2024). Effectiveness and safety of governor vessel acupuncture therapy for post-stroke cognitive impairment: a meta-analysis of randomized controlled trials. Ageing Res. Rev. 99:102355. doi: 10.1016/j.arr.2024.102355 38942201

[B30] Han Q. Zhang Y. Liu D. Wang Y. Feng Y. Yin X. . (2018). Disrupted local neural activity and functional connectivity in subjective tinnitus patients: evidence from resting-state fmri study. Neuroradiology 60, 1193–1201. doi: 10.1007/s00234-018-2087-0 30159629

[B31] Hilkens N. A. Casolla B. Leung T. W. de Leeuw F. E. (2024). Stroke. Lancet 403, 2820–2836. doi: 10.1016/S0140-6736(24)00642-1 38759664

[B32] Hoffmann M. (2020). “Clinical mentation evaluation,” in Prefrontal Network for Executive Control of Cognition and Comportment Including the Executive Control, Salience (ventral attention) and Semantic Appraisal (SAN) Networks. (Cham: Springer International Publsihing), 61–77. doi: 10.1007/978-3-030-46324-3_7

[B33] Hosseini A. Ispoglou S. Hayton T. Evans R. Wilson M. Sawlani V. . (2016). The role of hippocampal pathology in post-stroke cognitive impairment. J. Neurol. Neurosurg. Psychiatr. 87, 17–19. doi: 10.1136/jnnp-2016-315106.60

[B34] Hua Y. Geng Y. Liu S. Xia S. Liu Y. Cheng S. . (2024). Identification of specific abnormal brain functional activity and connectivity in cancer pain patients: a preliminary resting-state fmri study. J. Pain Res. 17, 3959–3971. doi: 10.2147/JPR.S470750 39600396 PMC11590652

[B35] Huang Y. Y. Chen S. D. Leng X. Y. Kuo K. Wang Z. T. Cui M. . (2022). Post-stroke cognitive impairment: epidemiology, risk factors, and management. J. Alzheimers. Dis. 86, 983–999. doi: 10.3233/JAD-215644 35147548

[B36] Irazoki E. Contreras-Somoza L. M. Toribio-Guzmán J. M. Jenaro-Río C. van der Roest H. Franco-Martín M. A. (2020). Technologies for cognitive training and cognitive rehabilitation for people with mild cognitive impairment and dementia. A systematic review. Front. Psychol. 11:648. doi: 10.3389/fpsyg.2020.00648 32373018 PMC7179695

[B37] Jiang C. Tao J. Huang J. Ye H. Chen L. (2014). Resting state functional magnetic resonance imaging of the hippocampus after ischemic stroke. Chin. J. Phys. Med. Rehab. 36, 517–522. doi: 10.3760/cma.j.issn.0254-1424.2014.07.005

[B38] Jung J. Laverick R. Nader K. Wilson M. Auer D. Rotshtein P. . (2020). Altered hippocampal functional connectivity patterns in patients with cognitive impairments following ischaemic stroke: a resting-state fmri study. Neuroimage 32:20219782. doi: 10.1101/2020.10.26.20219782 34266772 PMC8527045

[B39] Kalaria R. N. Akinyemi R. Ihara M. (2016). Stroke injury, cognitive impairment and vascular dementia. Biochim. Biophys. Acta 1862, 915–925. doi: 10.1016/j.bbadis.2016.01.015 26806700 PMC4827373

[B40] Kawada T. (2018). Montreal cognitive assessment (moca) and its memory tasks for detecting mild cognitive impairment. Neurol. Sci. 40:633. doi: 10.1007/s10072-018-3616-7 30357487

[B41] Klapwijk E. T. van den Bos W. Tamnes C. K. Raschle N. M. Mills K. L. (2021). Opportunities for increased reproducibility and replicability of developmental neuroimaging. Dev. Cogn. Neurosci. 47:100902. doi: 10.1016/j.dcn.2020.100902 33383554 PMC7779745

[B42] Kliper E. Assayag B. Korczyn A. Auriel E. Shopin L. Hallevi H. . (2016). Cognitive state following mild stroke: a matter of hippocampal mean diffusivity. Hippocampus 26:hipo22500. doi: 10.1002/hipo.22500 26222988

[B43] Kodama A. Suzuki Y. Sakuraba K. Kume Y. Ota H. (2022). The effect of deep micro vibrotactile stimulation on cognitive function of mild cognitive impairment and mild dementia. Int. J. Environ. Res. Public Health 19:ijerph19073803. doi: 10.3390/ijerph19073803 35409485 PMC8997479

[B44] Kong Q. Hodges S. Ursitti A. K. Reddy S. Zhu M. Kong J. (2024). Identifying potential scalp acupuncture targets for chronic pain and comorbid disorders using functional and anatomical connectivity of critical deep brain structures. Brain Behav. Immun. Integr. 5:100050. doi: 10.1016/j.bbii.2024.100050

[B45] Korostynski M. Hoinkis D. Piechota M. Golda S. Pera J. Slowik A. . (2021). Toll-like receptor 4-mediated cytokine synthesis and post-stroke depressive symptoms. Transl. Psychiatry 11:246. doi: 10.1038/s41398-021-01359-x 33903586 PMC8076201

[B46] Kuang X. Fan W. Hu J. Wu L. Yi W. Lu L. . (2021). Acupuncture for post-stroke cognitive impairment: a systematic review and meta-analysis. Acupunct. Med. 39, 577–588. doi: 10.1177/09645284211009542 34074151

[B47] Kuhnert M. Geier C. Elger C. E. Lehnertz K. (2012). Identifying important nodes in weighted functional brain networks: a comparison of different centrality approaches. Chaos 22:023142. doi: 10.1063/1.4729185 22757549

[B48] Kwon H. S. Lee D. Lee M. H. Yu S. Lim J. Yu K. . (2020). Post-stroke cognitive impairment as an independent predictor of ischemic stroke recurrence: picasso sub-study. J. Neurol. 267, 688–693. doi: 10.1007/s00415-019-09630-4 31720819

[B49] Lang M. Colby S. Ashby-Padial C. Bapna M. Jaimes C. Rincon S. . (2023). An imaging review of the hippocampus and its common pathologies. J. Neuroimag. 34, 25–25. doi: 10.1111/jon.13165 37872430

[B50] Lekoubou A. Nguyen C. Kwon M. Nyalundja A. D. Agrawal A. (2023). Post-stroke everything. Curr. Neurol. Neurosci. Rep. 23, 785–800. doi: 10.1007/s11910-023-01308-9 37837566

[B51] Lestari S. Mistivani I. Rumende C. Kusumaningsih W. (2017). Comparison between mini mental state examination (mmse) and montreal cognitive assessment indonesian version (moca-ina) as an early detection of cognitive impairments in post-stroke patients. J. Phys. Conf. Series 884:012153. doi: 10.1088/1742-6596/884/1/012153

[B52] Leung J. Lee G. Lam Y. Chan R. Wu J. (2011). The use of the digit span test in screening for cognitive impairment in acute medical inpatients. Int. Psychogeriatr. 23, 1569–1574. doi: 10.1017/S1041610211000792 21729426

[B53] Li M. Li Y. Zhang G. Chen J. Zhang J. Qi J. . (2015a). Acupuncture for ischemic stroke: cerebellar activation may be a central mechanism following deqi. Neural. Regen. Res. 10, 1997–2003. doi: 10.4103/1673-5374.172318 26889189 PMC4730825

[B54] Li M. Long C. Yang L. (2015b). Hippocampal-prefrontal circuit and disrupted functional connectivity in psychiatric and neurodegenerative disorders. Biomed. Res. Int. 2015:810548. doi: 10.1155/2015/810548 25918722 PMC4396015

[B55] Li N. Wang H. Liu H. Zhu L. Lyu Z. Qiu J. . (2023). The effects and mechanisms of acupuncture for post-stroke cognitive impairment: progress and prospects. Front. Neurosci. 17:1211044. doi: 10.3389/fnins.2023.1211044 37397457 PMC10309044

[B56] Li X. (2023). Study on the central mechanism of “Regulating spirit and benefiting intelligence” acupuncture method in improving cognitive function of post-stroke mild cognitive impairment patients based on resting-state fMRI. (dissertation). Tianjin: Tianjin University of Traditional Chinese Medicine.

[B57] Li Y. Liu B. Zhao T. Quan X. Han Y. Cheng Y. . (2023). Comparative study of extracellular vesicles derived from mesenchymal stem cells and brain endothelial cells attenuating blood-brain barrier permeability via regulating caveolin-1-dependent zo-1 and claudin-5 endocytosis in acute ischemic stroke. J. Nanobiotechnol. 21:70. doi: 10.1186/s12951-023-01828-z 36855156 PMC9976550

[B58] Li Z. Yin C. Shi H. Zhang C. Yang L. Du Y. (2024). Effect of acupuncture timing on functional impairment at 6 months post-onset in patients with first-ever stroke: a prospective cohort study. Zhongguo Zhen. Jiu. 44, 375–383. doi: 10.13703/j.0255-2930.20221205-0005 38621722

[B59] Li Z. Zhu M. Meng C. Lin H. Huang L. (2022). Predictive value of serum adiponectin and hemoglobin levels for vascular cognitive impairment in ischemic stroke patients. Pak. J. Med. Sci. 38, 705–710. doi: 10.12669/pjms.38.3.5204 35480518 PMC9002452

[B60] Liu J. Wang Q. Liu F. Song H. Liang X. Lin Z. . (2017). Altered functional connectivity in patients with post-stroke memory impairment: a resting fmri study. Exp. Ther. Med. 14, 1919–1928. doi: 10.3892/etm.2017.4751 28962104 PMC5609161

[B61] Liu R. Zhang K. Tong Q. Cui G. Ma W. Shen W. (2021). Acupuncture for post-stroke depression: a systematic review and meta-analysis. BMC Compl. Med. Ther. 21:109. doi: 10.1186/s12906-021-03277-3 PMC8017746 33794857

[B62] Liu Y. Chen F. Qin P. Zhao L. Li X. Han J. . (2023). Acupuncture treatment vs. Cognitive rehabilitation for post-stroke cognitive impairment: a systematic review and meta-analysis of randomized controlled trials. Front. Neurol. 14:1035125. doi: 10.3389/fneur.2023.1035125 36846126 PMC9946978

[B63] Lu J. Xing X. Qu J. Wu J. Zheng M. Hua X. . (2024). Alterations of contralesional hippocampal subfield volumes and relations to cognitive functions in patients with unilateral stroke. Brain Behav. 14:3645. doi: 10.1002/brb3.3645 39135280 PMC11319231

[B64] Lyrakos G. Ypofandi M. Tzanne P. (2014). Epa-1593 - psychometric and clinometric properties of the montreal cognitive assessment (moca) in a greek sample. Eur. Psychiatr. 29, 1–1. doi: 10.1016/S0924-9338(14)78748-6

[B65] Ma S. Huang H. Zhong Z. Zheng H. Li M. Yao L. . (2022). Effect of acupuncture on brain regions modulation of mild cognitive impairment: a meta-analysis of functional magnetic resonance imaging studies. Front. Aging Neurosci. 14:914049. doi: 10.3389/fnagi.2022.914049 36212046 PMC9540390

[B66] Ma S. Wang L. Su X. Yang N. Huang J. Lin L. . (2020). Acupuncture improves white matter perfusion and integrity in rat model of vascular dementia: an mri-based imaging study. Front. Aging Neurosci. 12:582904. doi: 10.3389/fnagi.2020.582904 33328963 PMC7719770

[B67] Madsen T. E. Guo D. (2020). Sex differences in modifiable stroke risk factors: the next step in personalized stroke prevention. Neurology 95, 891–892. doi: 10.1212/WNL.0000000000010983 33067407

[B68] Malik Y. A. Awad T. A. Abdalla M. Yagi S. Alhazmi H. A. Ahsan W. . (2022). Chalcone scaffolds exhibiting acetylcholinesterase enzyme inhibition: mechanistic and computational investigations. Molecules 27:27103181. doi: 10.3390/molecules27103181 35630658 PMC9145706

[B69] Mattson M. P. (2000). Apoptosis in neurodegenerative disorders. Nat. Rev. Mol. Cell Biol. 1, 120–129. doi: 10.1038/35040009 11253364

[B70] Meng N. Shi S. Su Y. (2016). Proton magnetic resonance spectroscopy as a diagnostic biomarker in mild cognitive impairment following stroke in acute phase. NeuroReport 27:559. doi: 10.1097/WNR.0000000000000555 26981713

[B71] Mijajlović M. D. Pavlović A. Brainin M. Heiss W. Quinn T. J. Ihle-Hansen H. B. . (2017). Post-stroke dementia - a comprehensive review. BMC Med. 15:11. doi: 10.1186/s12916-017-0779-7 28095900 PMC5241961

[B72] Moher D. Liberati A. Tetzlaff J. Altman D. G. (2009). Preferred reporting items for systematic reviews and meta-analyses: the prisma statement. Ann. Intern. Med. 151:W64. doi: 10.7326/0003-4819-151-4-200908180-00135 19622511

[B73] Pendlebury S. Markwick A. Jager C. Zamboni G. Wilcock G. Rothwell P. (2012). Differences in cognitive profile between tia, stroke and elderly memory research subjects: a comparison of the mmse and moca. Cerebrovasc. Dis. 34, 48–54. doi: 10.1159/000338905 22759627

[B74] Pendlebury S. T. Rothwell P. M. (2009). Prevalence, incidence, and factors associated with pre-stroke and post-stroke dementia: a systematic review and meta-analysis. Lancet Neurol. 8, 1006–1018. doi: 10.1016/S1474-4422(09)70236-4 19782001

[B75] Qin S. Zhang Z. Zhao Y. Liu J. Qiu J. Gong Y. . (2022). The impact of acupuncture on neuroplasticity after ischemic stroke: a literature review and perspectives. Front. Cell. Neurosci. 16:817732. doi: 10.3389/fncel.2022.817732 36439200 PMC9685811

[B76] Qiu X. Yao X. Han S. Wu Y. Ou Z. Li T. . (2021). Acupuncture reduces the risk of dysphagia in stroke patients: a propensity score-matched cohort study. Front. Neurosci. 15:791964. doi: 10.3389/fnins.2021.791964 35069105 PMC8770751

[B77] Sang L. Liu C. Wang L. Zhang J. Zhang Y. Li P. . (2020). Disrupted brain structural connectivity network in subcortical ischemic vascular cognitive impairment with no dementia. Front. Aging Neurosci. 12:6. doi: 10.3389/fnagi.2020.00006 32063840 PMC7000429

[B78] Shi Y. Zhang S. Li Q. Liu Z. Guo S. Yang J. . (2016). A study of the brain functional network of deqi via acupuncturing stimulation at bl40 by rs-fmri. Comp. Ther. Med. 25, 71–77. doi: 10.1016/j.ctim.2016.01.004 27062952

[B79] Shishkina G. T. Kalinina T. S. Gulyaeva N. V. Lanshakov D. A. Dygalo N. N. (2021). Changes in gene expression and neuroinflammation in the hippocampus after focal brain ischemia: involvement in the long-term cognitive and mental disorders. Biochemistry 86, 657–666. doi: 10.1134/S0006297921060043 34225589

[B80] Smagula S. Karim H. Rangarajan A. Santos F. P. Wood S. Santini T. . (2018). Association of hippocampal substructure resting-state functional connectivity with memory performance in older adults. Am. J. Ger. Psychiatr. 26, 690–699. doi: 10.1016/j.jagp.2018.03.003 29628321 PMC5993618

[B81] Snowball A. Tachtsidis I. Popescu T. Thompson J. Delazer M. Zamarian L. . (2013). Long-term enhancement of brain function and cognition using cognitive training and brain stimulation. Curr. Biol. 23, 987–992. doi: 10.1016/j.cub.2013.04.045 23684971 PMC3675670

[B82] Stinear C. M. Lang C. E. Zeiler S. Byblow W. D. (2020). Advances and challenges in stroke rehabilitation. Lancet Neurol. 19, 348–360. doi: 10.1016/S1474-4422(19)30415-6 32004440

[B83] Su J. Fan L. Li M. Qiu Q. Liu Y. (2024). Therapeutic effect of Xingnao Kaiqiao acupuncture combined with repetitive transcranial magnetic stimulation on post-stroke cognitive impairment. Shizhen Nat. Med. Mat. Med. 35, 3421–3423. doi: 10.3969/j.issn.1008-0805.2024.15.18

[B84] Su M. (2016). Clinical study on acupuncture combined with nimodipine in the treatment of mild cognitive impairment after acute cerebral infarction (phlegm and blood stasis syndrome). (Master's thesis). Fuzhou: Fujian University of Traditional Chinese Medicine.

[B85] Sun J. Tan L. Yu J. (2014). Post-stroke cognitive impairment: epidemiology, mechanisms and management. Ann. Transl. Med. 2:80. doi: 10.3978/j.issn.2305-5839.2014.08.05 25333055 PMC4200648

[B86] Swartz R. H. Bayley M. Lanctôt K. L. Murray B. J. Cayley M. L. Lien K. . (2016). Post-stroke depression, obstructive sleep apnea, and cognitive impairment: rationale for, and barriers to, routine screening. Int. J. Stroke 11, 509–518. doi: 10.1177/1747493016641968 27073189

[B87] Tsai C. Liao W. Wu H. Chang C. Chen W. Hsieh C. (2024). Acupuncture improves neurological function and anti-inflammatory effect in patients with acute ischemic stroke: a double-blinded randomized controlled trial. Complement. Ther. Med. 82:103049. doi: 10.1016/j.ctim.2024.103049 38729273

[B88] Tumati S. Martens S. Aleman A. (2013). Magnetic resonance spectroscopy in mild cognitive impairment: systematic review and meta-analysis. Neurosci. Biobehav. Rev. 37, 2571–2586. doi: 10.1016/j.neubiorev.2013.08.004 23969177

[B89] Veldsman M. Cheng H. Ji F. Werden E. Khlif M. Ng K. . (2020). Degeneration of structural brain networks is associated with cognitive decline after ischaemic stroke. Brain Commun. 2:fcaa155. doi: 10.1093/braincomms/fcaa155 33376984 PMC7751023

[B90] Wahl A. (2018). State-of-the-art techniques to causally link neural plasticity to functional recovery in experimental stroke research. Neural. Plast. 2018:3846593. doi: 10.1155/2018/3846593 29977279 PMC5994266

[B91] Wang F. Gao S. Yang L. (2021). Observation on the efficacy of acupuncture combined with cognitive training. Shanghai J. Acupunct. Moxibustion 40, 795–800. doi: 10.13460/j.issn.1005-0957.2021.07.0795

[B92] Wang F. Liang H. Chen S. Huang J. Lin Q. (2014). Study on magnetic resonance spectroscopy. Chin. J. Trad. Chin. Med. Emerg. 23, 1928–1930. doi: 10.3969/j.issn.1004-745X.2014.10.072

[B93] Wang J. (2020). Expert consensus on the prevention and treatment of post-stroke cognitive impairment in China. Chin. J. Stroke 15, 158–166. doi: 10.1007/978-981-10-1433-8_2

[B94] Wang L. Yang J. Lin L. Huang J. Wang X. Su X. . (2020). Acupuncture attenuates inflammation in microglia of vascular dementia rats by inhibiting mir-93-mediated tlr4/myd88/nf-κb signaling pathway. Oxid. Med. Cell. Longev. 2020:8253904. doi: 10.1155/2020/8253904 32850002 PMC7441436

[B95] Wang R. (2021). Study on brain functional network of patients with post-stroke cognitive impairment treated by acupuncture. (Master's thesis). Nanchong: North Sichuan Medical College.

[B96] Wang X. Wei W. Bai Y. Shen Y. Zhang G. Meng N. . (2023). Intrinsic brain activity alterations in patients with parkinson's disease. Neurosci. Lett. 809:137298. doi: 10.1016/j.neulet.2023.137298 37196973

[B97] Wang Y. Chen H. Wang C. Liu J. Miao P. Wei Y. . (2024a). Static and dynamic interactions within the triple-network model in stroke patients with multidomain cognitive impairments. Neuroimage 43:103655. doi: 10.1016/j.nicl.2024.103655 39146837 PMC11367478

[B98] Wang Y. Chen W. Lee C. Shen Y. Lan C. Liu G. . (2022a). Combinations of scalp acupuncture location for the treatment of post-stroke hemiparesis: a systematic review and apriori algorithm-based association rule analysis. Front. Neurosci. 16:956854. doi: 10.3389/fnins.2022.956854 35992903 PMC9389219

[B99] Wang Y. Liu Y. Zhang Z. Qiao X. Li Y. Ren L. . (2024b). Influence of acupuncture intensity on analgesic effects in aa rat models. Front. Bioeng. Biotechnol. 12:1502535. doi: 10.3389/fbioe.2024.1502535 39723129 PMC11668573

[B100] Wang Y. Wang L. Wang Y. Lu M. Xu L. Liu R. . (2022b). Sensorimotor responses in post-stroke hemiplegic patients modulated by acupuncture at yanglingquan (gb34): a fmri study using intersubject functional correlation (isfc) analysis. Front. Neurol. 13:900520. doi: 10.3389/fneur.2022.900520 35734477 PMC9208550

[B101] Wang Y. Xu N. Wang R. Zai W. (2022c). Systematic review and network meta-analysis of effects of noninvasive brain stimulation on post-stroke cognitive impairment. Front. Neurosci. 16:1082383. doi: 10.3389/fnins.2022.1082383 36643019 PMC9832390

[B102] Wang Z. Sun Z. Zhang M. Xiong K. Zhou F. (2022d). Systematic review and meta-analysis of acupuncture in the treatment of cognitive impairment after stroke. Medicine 101:e30461. doi: 10.1097/MD.0000000000030461 36254056 PMC9575739

[B103] Wei X. Chen H. Guo C. Tan W. Zhan S. (2021). The instant and sustained effect of electroacupuncture in postgraduate students with depression: an fmri study. Neuropsychiatr. Dis. Treat. 17, 873–883. doi: 10.2147/NDT.S307083 33776442 PMC7989050

[B104] Whitwell J. Przybelski S. Weigand S. Knopman D. Boeve B. Petersen R. . (2007). 3d maps from multiple mri illustrate changing atrophy patterns as subjects progress from mild cognitive impairment to alzheimer's disease. Brain J. Neurol. 130,1777–1786. doi: 10.1093/brain/awm112 17533169 PMC2752411

[B105] Whyte E. M. Lenze E. J. Butters M. Skidmore E. Koenig K. Dew M. A. . (2008). An open-label pilot study of acetylcholinesterase inhibitors to promote functional recovery in elderly cognitively impaired stroke patients. Cerebrovasc. Dis. 26, 317–321. doi: 10.1159/000149580 18667813 PMC2914451

[B106] Winstein C. J. Stein J. Arena R. Bates B. Cherney L. R. Cramer S. C. . (2016). Guidelines for adult stroke rehabilitation and recovery: a guideline for healthcare professionals from the american heart association/american stroke association. Stroke 47, e98–e169. doi: 10.1161/STR.0000000000000098 27145936

[B107] Wu W. Song C. Yang Y. Hu Y. Lin H. (2024). Acupuncture for cognitive impairment after stroke: a systematic review and meta-analysis. Heliyon 10:e30522. doi: 10.1016/j.heliyon.2024.e30522 38765166 PMC11098789

[B108] Xiang A. Chen M. Qin C. Rong J. Wang C. Shen X. . (2021). Frequency-specific blood oxygen level dependent oscillations associated with pain relief from ankle acupuncture in patients with chronic low back pain. Front. Neurosci. 15:786490. doi: 10.3389/fnins.2021.786490 34949986 PMC8688988

[B109] Xiao W. Yu J. (2024). Effects of acupuncture at Neiguan, Baihui, and Sishencong on post-stroke cognitive impairment and brain network properties. Modern Health 24, 1472–1475.

[B110] Xin-Xian Z. (2012). Research of Memory Impairment Following Subcortical Single Strokes in Four Different Parts. Hangzhou: Clinical Education of General Practice.

[B111] Yang S. Jiang C. Ye H. Tao J. Huang J. Gao Y. . (2014). Effect of integrated cognitive therapy on hippocampal functional connectivity patterns in stroke patients with cognitive dysfunction: a resting-state fmri study. eCAM 2014:962304. doi: 10.1155/2014/962304 25548595 PMC4274659

[B112] Yang T. Liu W. He J. Gui C. Meng L. Xu L. . (2024). The cognitive effect of non-invasive brain stimulation combined with cognitive training in alzheimer's disease and mild cognitive impairment: a systematic review and meta-analysis. Alzheimers. Res. Ther. 16:140. doi: 10.1186/s13195-024-01505-9 38937842 PMC11212379

[B113] Yang X. Shi G. Li Q. Zhang Z. Xu Q. Liu C. (2013). Characterization of deqi sensation and acupuncture effect. Evid. Based Complement. Alternat. Med. 2013:319734. doi: 10.1155/2013/319734 23864884 PMC3705793

[B114] Yang X. Shi L. Ran D. Li M. Qin C. An Z. (2022). The treatment of post-stroke dysarthria with a combination of different acupuncture types and language rehabilitation training: a systematic review and network meta-analysis. Ann. Transl. Med. 10:1281. doi: 10.21037/atm-22-5583 36618810 PMC9816828

[B115] Yin Z. Wang Z. Li Y. Zhou J. Chen Z. Xia M. . (2023a). Neuroimaging studies of acupuncture on alzheimer's disease: a systematic review. BMC Complement. Med. Ther. 23:63. doi: 10.1186/s12906-023-03888-y 36823586 PMC9948384

[B116] Yin Z. Zhou J. Xia M. Chen Z. Li Y. Zhang X. . (2023b). Acupuncture on mild cognitive impairment: a systematic review of neuroimaging studies. Front. Aging Neurosci. 15:1007436. doi: 10.3389/fnagi.2023.1007436 36875696 PMC9975578

[B117] Yu P. Dong R. Wang X. Tang Y. Liu Y. Wang C. . (2024). Neuroimaging of motor recovery after ischemic stroke - functional reorganization of motor network. Neuroimage Clin. 43:103636. doi: 10.1016/j.nicl.2024.103636 38950504 PMC11267109

[B118] Yu Y. Cheng M. Ma M. Liu L. (2021). Effect of Bo's abdominal acupuncture on resting-state fMRI in patients with post-stroke cognitive impairment. Shanghai J. Acupunct. Moxibustion 40, 1293–1298. doi: 10.13460/j.issn.1005-0957.2021.11.1293

[B119] Yuan D. Tian H. Zhou Y. Wu J. Sun T. Xiao Z. . (2021). Acupoint-brain (acubrain) mapping: common and distinct cortical language regions activated by focused ultrasound stimulation on two language-relevant acupoints. Brain Lang. 215:104920. doi: 10.1016/j.bandl.2021.104920 33561785

[B120] Yue X. Li Z. Li Y. Gao J. Han H. Zhang G. . (2023). Altered static and dynamic functional network connectivity in post-stroke cognitive impairment. Neurosci. Lett. 799:137097. doi: 10.1016/j.neulet.2023.137097 36716911

[B121] Zhang J. Cai X. Wang Y. Zheng Y. Qu S. Zhang Z. . (2019). Different brain activation after acupuncture at combined acupoints and single acupoint in hypertension patients: an rs-fmri study based on reho analysis. Evid. Based Complement. Alternat. Med. 2019:5262896. doi: 10.1155/2019/5262896 30719061 PMC6335668

[B122] Zhang J. Wei R. Yang H. Shen Y. Zheng J. (2020). Scalp acupuncture combined with computer-assisted training for post-cerebral infarction cognitive impairment in older adults: a magnetic resonance spectroscopic imaging study. Chin. J. Gerontol. 40, 4067–4070. doi: 10.3969/j.issn.1005-9202.2020.19.011

[B123] Zhang S. Wang Y. Zhang C. Zhang C. Xiao P. Li Q. . (2022). Effect of interactive dynamic scalp acupuncture on post-stroke cognitive function, depression, and anxiety: a multicenter, randomized, controlled trial. Chin. J. Integr. Med. 28, 106–115. doi: 10.1007/s11655-021-3338-1 34874523

[B124] Zhang Y. Tang Y. Peng Y. Yan Z. Zhou J. Yue Z. (2024). Acupuncture, an effective treatment for post-stroke neurologic dysfunction. Brain Res. Bull. 215:111035. doi: 10.1016/j.brainresbull.2024.111035 39069104

[B125] Zhen D. Xia W. Yi Z. Q. Zhao P. W. Zhong J. G. Shi H. C. . (2018). Alterations of brain local functional connectivity in amnestic mild cognitive impairment. Transl. Neurodegener. 7:26. doi: 10.1186/s40035-018-0134-8 30443345 PMC6220503

[B126] Zheng Y. Qin Z. Tsoi B. Shen J. Zhang Z. (2020). Electroacupuncture on trigeminal nerve-innervated acupoints ameliorates poststroke cognitive impairment in rats with middle cerebral artery occlusion: involvement of neuroprotection and synaptic plasticity. Neural. Plast. 2020:8818328. doi: 10.1155/2020/8818328 32963517 PMC7492933

[B127] Zinman J. Kapoor A. Si K. Sujanthan S. Southwell A. Cayley M. L. . (2023). Men are at higher risk of screening positive for vascular cognitive impairment compared to women after stroke and transient ischemic attack. J. Alzheimers. Dis. 94, 89–94. doi: 10.3233/JAD-230021 37212109

