# Supplementary material for: Neuroimaging evidence of acupuncture in cognitive impairment following ischemic stroke: a systematic review
Source: Front Neurosci. 2026 Jan 12;19:1629305. doi: 10.3389/fnins.2025.1629305 (PMC12832758; doi:10.3389/fnins.2025.1629305)
Supplement: Supplementary file 1 [file Data_Sheet_1.docx]

Supplementary Materials

Supplementary Table S1. Neuroimaging Detailed Results After Sustained Acupuncture Treatment

| Study | Type of A patient | Type of B patient | (A)Intervention group | (B)Control group | Imaging modality | Analytical approaches | MRIScanner | Neuroimaging results |
| --- | --- | --- | --- | --- | --- | --- | --- | --- |
| Wei Xiao | PISCI | PISCI | Acupuncture | Sham acupuncture | rs-fMRI | DC | / | Brain regions with increased degree centrality in the control group after treatment compared to before treatment: left dorsolateral superior frontal gyrus, right inferior occipital gyrus, left inferior parietal angular gyrus, right transverse temporal gyrus; brain regions with decreased degree centrality: right middle frontal gyrus, right opercular part of inferior frontal gyrus, left middle occipital gyrus.  Brain regions with increased degree centrality in the intervention group after treatment compared to before treatment: left parahippocampal gyrus, left posterior cingulate gyrus, left fusiform gyrus, left transverse temporal gyrus; brain regions with decreased degree centrality: right angular gyrus.  Brain regions with increased degree centrality in both groups after treatment: left lingual gyrus, right lingual gyrus, left inferior occipital gyrus. Brain regions with decreased degree centrality: right orbital middle frontal gyrus.  Correlation analysis with neuropsychological scales revealed that the degree centrality AUC of the left inferior occipital gyrus after treatment in the intervention group was negatively correlated with MoCA scale scores. |
| Xiayu Li | PISCI | HC | Conventional therapy&"Regulating Mind and Enlightening Wisdom" acupuncture | / | rs-fMRI | FALFF,ReHo,DC | 3.0 T | Compared with the control group, the experimental group showed decreased fALFF values in the right precuneus, decreased ReHo values in the left cerebellar peduncle II, and decreased DC values in the right precuneus.  Compared with pre-acupuncture treatment, the experimental group demonstrated increased fALFF values in the left fusiform gyrus and decreased fALFF values in the right superior occipital gyrus after acupuncture treatment, while no significant differences were observed in ReHo or DC.  Correlation analysis revealed no significant associations between fALFF, ReHo, or DC values in the identified brain regions and neuropsychological scale scores. |
| Fei Wang | PISCI | PISCI | Acpuncture&Cognitive Training | Cognitive Training | rs-fMRI | FC | 3.0 T | Compared to before treatment, the fMRI of patients in the intervention group showed enhanced functional connectivity between the left hippocampus and the right inferior frontal gyrus and right middle frontal gyrus, as well as between the right hippocampus and the left middle frontal gyrus, left inferior frontal gyrus, left superior frontal gyrus, and left parietal lobe. |
| Ran Wang | PISCI | HC | Acupuncture | / | rs-fMRI | Brain Function Network | 3.0T | Compared to the normal control group, the intervention group patients showed reductions in normalized clustering coefficient, clustering coefficient (C), and local efficiency (Eloc). Comparing the intervention group patients after treatment to before treatment, there were no statistically significant differences in global brain network attributes. Regarding nodal attributes, compared to the normal group, the intervention group patients before treatment showed abnormal node centrality mainly involving the default mode network (DMN) and executive control network (ECN); all brain regions with differences showed increased nodal local efficiency. Comparing the intervention group patients after acupuncture treatment to before treatment, bilateral lingual gyri and left inferior occipital gyrus showed increased node centrality; the left parahippocampal gyrus, right inferior occipital gyrus, left fusiform gyrus, and left transverse gyrus showed increased nodal local efficiency, while the right angular gyrus showed decreased nodal local efficiency.  Correlation analysis with neuropsychological scales found that: the AUC of nodal local efficiency in the right orbital superior frontal gyrus before treatment in the intervention group was positively correlated with MoCA scale scores; the AUC of degree centrality in the left inferior occipital gyrus after acupuncture treatment in the intervention group was negatively correlated with MoCA scale scores. |
| Yanli Yu | PISCI | PISCI | Bo’s Abdominal Acupuncture&Cognitive Training | Conventional therapy& Cognitive Training | rs-fMRI | ALFF | / | The intervention group showed increased ALFF values in the right parahippocampal gyrus, left thalamus, right insula, and left anterior cingulate cortex. Decreased ALFF values were observed in the left posterior cerebellum, left inferior temporal gyrus, right inferior temporal gyrus, left inferior frontal gyrus, left middle temporal gyrus, left inferior occipital gyrus, and left superior parietal lobule. |
| Jianbo Zhang | PISCI | PISCI | Scalp acupuncture&Computer-Assisted Training | Computer-Assisted Training | MRS | Metabolic Ration (area of cerebral infarction) | 3.0T | After treatment, both groups showed a significant increase in NAA/Cr and a significant decrease in Cho/Cr and MI/Cr compared to before treatment. Moreover, the intervention group exhibited more pronounced changes in NAA/Cr and Cho/Cr compared to the control group. |
| Mengrun Su | PISCI | PISCI | Acupuncture&nimodipine | Nimodipine | MRS | Metabolic Ration (Bilateral frontal lobe white matter) | 3.0T | Post-treatment NAA/Cr levels were higher than pre-treatment levels. In the control group, there was a significant difference in left NAA/Cr levels before and after treatment, while the right NAA/Cr levels showed a statistically significant difference. In the intervention group, there was a significant difference in NAA/Cr levels on both sides before and after treatment. MI/Cr levels decreased post-treatment. In the control group, there was a statistically significant difference in left MI/Cr levels before and after treatment, while the right MI/Cr levels showed a significant difference. In the intervention group, there was a significant. |
| Fang Wang | PISCI | PISCI | Acupuncture&nimodipine | Nimodipine | MRS | Metabolic Ration(hippocampal region) | 3.0 T | Post-treatment, NAA/Cr ratios increased in both groups compared to pre-treatment levels, while Cho/Cr ratios decreased. In the intervention group, the increase in NAA/Cr ratio was more pronounced compared to the control group, and the decrease in Cho/Cr ratio was also more significant than in the control group. |

Note: PISCI, post-ischemic stroke cognitive impairment; HC, healthy controls; rs-fMRI, resting-state functional magnetic resonance imaging; MRS, magnetic resonance spectroscopy; DC, degree centrality; fALFF, fractional amplitude of low-frequency fluctuations; ReHo, regional homogeneity; FC, functional connectivity.

Supplementary Figure S1. Top 10 Most Frequently Used Acupoints in Included Studies

Note: The bar chart shows the 10 most frequently used acupoints across all eight included studies. GV20 (Baihui) was the most commonly used acupoint (75%), followed by EX-HN1 (Sishencong, 62.5%).
